# Phytotoxicity Mitigation and Malachite Green Removal from Wastewater Using Superparamagnetic Activated Carbon

**DOI:** 10.1021/acsomega.5c04838

**Published:** 2025-07-21

**Authors:** Sujesh Sudarsan, Gokulakrishnan Murugesan, Thivaharan Varadavenkatesan, Ramesh Vinayagam, Raja Selvaraj

**Affiliations:** † Department of Chemical Engineering, Manipal Institute of Technology, Manipal Academy of Higher Education, Manipal 576104, Karnataka, India; ‡ Department of Biotechnology, M.S. Ramaiah Institute of Technology, Bengaluru 560054, Karnataka, India; § Department of Biotechnology, Manipal Institute of Technology, Manipal Academy of Higher Education, Manipal 576104, Karnataka, India

## Abstract

This study addresses the removal of malachite green (MG) dye from water utilizing magnetic activated carbon prepared from flowers of *Spathodea campanulata* (SCMAC). The prepared SCMAC displayed an exceptional specific surface area (1012.9 m^2^/g) with mesopores (2.97 nm) and confirmed the superparamagnetism even after MG adsorption. FESEM revealed a highly porous structure with uniformly distributed Fe_3_O_4_ nanoparticles (40.84 nm). XPS analysis revealed shifts in the C 1s, O 1s, and Fe 2p binding energies after MG adsorption, indicating the involvement of π–π interactions, hydrogen bonding, and surface complexation between MG molecules and the oxygenated functional groups and Fe^2+^/Fe^3+^ sites on SCMAC. Batch adsorption studies revealed optimal conditions for MG removal (pH 4, 15 mg/L MG dye, 0.15 g/L SCMAC). Adsorption kinetic data obeyed the pseudo-second-order kinetics, and the Freundlich isotherm fitted well. The thermodynamic analysis demonstrated endothermic and spontaneous adsorption. Spiking studies demonstrated the practical applicability of SCMAC in industrial groundwater, achieving an adsorption capacity of 82.54 mg/g. Desorption studies showed 66.3% efficiency retention after six cycles. Phytotoxicity assessments revealed that after MG adsorption treatment with SCMAC, the germination index of *Solanum lycopersicum* seeds increased to 71.42%, underscoring its practical and ecological benefits. These findings establish the synthesized SCMAC as an efficient, sustainable, and reusable adsorbent, offering a practical and environmentally safe solution for wastewater treatment and remediation.

## Graphical abstract

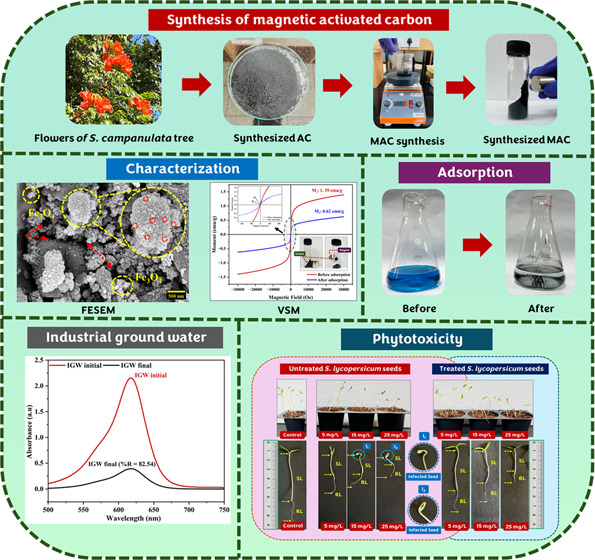

## 1 Introduction

Environmental contamination is one of the most pressing global crises of the 21st century, posing serious threats to human health, ecosystems, and economic stability. A major facet of this problem is water pollution, which is largely driven by industrial activities. Among these, the textile industry is a significant contributor, releasing large quantities of hazardous dyes and chemicals into water bodies. Malachite green (MG), a widely used cationic dye in the textile, aquaculture, and leather industries, has become a particular concern due to its high toxicity, nonbiodegradability, and resistance to conventional treatment methods.^1^ Its persistence in aquatic ecosystems not only disrupts food chains and harms aquatic organisms but also poses significant health risks to humans, including respiratory problems and carcinogenic effects, through exposure to contaminated water.^2^ These environmental and public health concerns underscore the urgent need for efficient and sustainable treatment strategies capable of effectively removing MG dye from contaminated water sources.

Conventional methods for dye removal encompass physical, chemical, and biological approaches, each with its own set of advantages and limitations. Physical methods, such as membrane filtration, can effectively separate dyes from wastewater but are often limited by high operational costs, energy demands, and membrane fouling.^3^ Chemical techniques, such as oxidation and coagulation break down dyes; however, they typically require expensive reagents and may produce harmful byproducts or large volumes of sludge.^4^ Biological methods, while environmentally friendly and economical, are generally unsuitable for treating complex synthetic dyes like MG and are highly sensitive to changes in environmental conditions.^5^

In contrast, adsorption has emerged as a promising alternative due to its operational simplicity, cost-effectiveness, minimal sludge generation, and high removal efficiency for dyes such as MG. A wide range of adsorbents, including agricultural byproducts, biochar,^6^ zeolites, and metal organic frameworks (MOFs), have been investigated for this purpose.^7^ However, the practical application of many of these materials is limited by challenges such as high production costs, poor structural stability in aqueous environments, insufficient reusability, and difficulties in large-scale synthesis and standardization.^8^ Activated carbon (AC) is widely favored due to its high surface area and porous structure, which offer abundant active sites for pollutant adsorption.^6^ Despite its proven effectiveness, AC presents operational challenges related to its separation and recovery from treated water, often necessitating additional filtration steps that increase treatment costs and complexity.^9^ These limitations have driven the development of advanced materials^10^ such as magnetic activated carbon (MAC), which incorporates magnetic components into the AC matrix. This magnetic modification enables rapid and efficient recovery of the adsorbent using an external magnetic field, eliminating the need for conventional filtration. Moreover, MAC retains the high adsorption capacity of AC while offering improved reusability and ease of handling, making it a practical and cost-effective solution for dye removal from wastewater.^3^

However, despite these advantages, only a limited number of studies have explored the application of MAC for MG removal, and many of the reported synthesis methods remain energy- and resource-intensive. For instance, Pang et al. synthesized iron-modified sugar cane bagasse AC via chemical impregnation followed by high-temperature calcination at 550 °C under nitrogen gas flow, which increases energy consumption and operational cost.^11^ The use of multiple chemical reagents, including iron nitrate [Fe­(NO_3_)_3_] and potassium hydroxide [KOH], and a two-step thermal treatment further complicates the process and limits scalability. Furthermore, the study did not investigate the material’s reusability or long-term stability—an important consideration for practical deployment, especially when the adsorbent or catalyst is expected to be reused across multiple treatment cycles.

Similarly, Guo et al. synthesized Fe–Mg bimetallic MAC using peanut shells through a multistep carbonization process involving sequential thermal treatments under nitrogen and carbon dioxide atmospheres, reaching temperatures as high as 800 °C.^12^ The method required a high dosage of activating agents, including 15 g of magnesium chloride hexahydrate (MgCl_2_·6H_2_O) and 10 g of ferric chloride hexahydrate (FeCl_3_·6H_2_O), along with prolonged processing time, resulting in elevated energy consumption and chemical input. The resulting adsorbent exhibited a relatively low specific surface area (SSA) of 633.35 m^2^/g. Furthermore, regeneration using ethanol showed a substantial decline in performance, with only 30.85% of the original capacity retained after the fourth cycle, indicating poor reusability and the potential need for energy-intensive thermal regeneration. In addition, the study did not evaluate the material’s performance in spiked real water matrices, which is a critical step for determining its practical applicability. Phytotoxicity analysis, which is crucial for assessing the ecological safety of treated effluents, was also not conducted, leaving the environmental compatibility of the material unaddressed. These limitations are further compounded by the relatively low adsorption capacities reported for MACs synthesized from various biomass sources. For instance, Datta et al. reported a maximum adsorption capacity of 36.36 mg/g using Fe_3_O_4_-loaded AC prepared from sawdust.^13^ Similarly, Bonyadi et al. synthesized a magnetic biocomposite using Fe_3_O_4_ and sawdust carbon, which exhibited a moderate adsorption capacity of 41.66 mg/g.^14^ These relatively low capacities underscore the need to explore alternative biomass precursors capable of producing MACs with enhanced MG adsorption performance.

*Spathodea campanulata* (African tulip tree), a widely cultivated ornamental species in tropical and subtropical regions such as India, presents an abundant yet underutilized source of floral biomass suitable for AC production.^7^ Its large seasonal blooms often lead to unmanaged organic litter in urban areas, posing disposal challenges.^15^ Unlike agricultural and forestry residues, which are frequently diverted for uses such as composting, fodder, or energy, *S. campanulata* flowers are typically discarded, making them a sustainable, low-cost, and noncompetitive precursor for carbon synthesis. Importantly, valorizing this biomass not only contributes to urban green waste management but also supports circular bioeconomy initiatives.

Despite these advantages, only a limited number of studies have examined the adsorption potential of *S. campanulata*-derived materials. For instance, Teweldebrihan and Dinka investigated AC derived from the plant’s stems for Cr­(VI) removal and reported a relatively low adsorption capacity of 10.65 mg/g.^16^ Ravikumar and King explored powdered leaf biomass for the removal of Congo red (CR) and crystal violet (CV) dyes, with maximum adsorption capacities of 11.73 mg/g and 12.65 mg/g, respectively.^17,18^ However, these studies employed untreated or nonmagnetized biosorbents, which likely limited adsorption efficiency due to reduced surface area and restricted accessibility to active sites. Furthermore, key material attributes such as BET surface area, pore structure, and functional groups were not characterized, hindering mechanistic understanding and performance optimization. A more recent study by Dimbo et al. synthesized orthophosphoric acid (H_3_PO_4_) activated AC from *S. campanulata* stems, achieving a high surface area (1054 m^2^/g) and efficient methylene blue (MB) dye removal.^15^ Nonetheless, the study had notable limitations: the adsorbent lacked magnetic functionalization for easy separation and reuse; regeneration studies to assess material longevity were not conducted; adsorption was tested only in synthetic dye solutions without evaluation in real water matrices; and ecological safety was not assessed via phytotoxicity testing. Moreover, advanced characterizations such as XPS, EDS, and XRD were not performed, and the synthesis process involved higher energy consumption due to a pyrolysis temperature of 500 °C. Our team has recently published an article highlighting the *S. campanulata* flower as an inexpensive precursor for AC preparation to remove CR dye.^7^

Addressing these limitations, the present study focuses on the use of *S. campanulata* flowers as a novel and eco-friendly precursor for synthesizing MAC aimed at removing MG dye from aqueous systems. The synthesis utilizes H_3_PO_4_ as an activating agent and operates at a milder temperature of 400 °C without requiring an inert atmosphere, thereby reducing energy input and avoiding the use of corrosive or hazardous chemicals such as ZnCl_2_, KOH, or H_2_SO_4_. To ensure practical viability, the study includes adsorption kinetics and isotherm modeling, regeneration experiments to assess reusability, spiking tests in real water matrices to evaluate operational robustness, and phytotoxicity assays to confirm ecological safety of the treated water. Collectively, this work provides a sustainable and scalable approach for effective dye remediation from wastewater, addressing critical shortcomings of earlier studies.

## 2 Results and Discussion

### 2.1 Morphology and Elemental Features of *S. campanulata*

The FESEM analysis highlights significant morphological differences in SCMAC before and after MG adsorption. Before adsorption (Figure 1a), the SCMAC surface exhibited a highly porous and irregular structure,^19^ characterized by numerous cavities and voids^20^ (depicted with red dotted lines in Figure 1a). Additionally, the magnified FESEM image demonstrated the presence of aggregates containing spherical Fe_3_O_4_ nanoparticles, formed during the activation and magnetic modification process. These nanoparticles, with an average size of 40.84 ± 2.47 nm (Figure 1a inset), are uniformly distributed across the SCMAC surface, further emphasizing its potential for convenient separation and reuse. In comparison, the study by Ebadollahzadeh and Zabihi reported magnetic wood-based AC with sizes smaller than 50 nm and a spherical shape.^21^ After adsorption, the porous structure becomes less pronounced and smoother with partial or complete blockage of the pores due to the accumulation of MG molecules on the surface (Figure 1b). These findings are concordant with the results of Jafari Harandi et al., who documented a significant reduction in the apparent surface porosity of the synthesized nanocomposite after adsorbing reactive green KE-4BD dye onto AC derived from coconut husk.^22^

**Figure 1:**
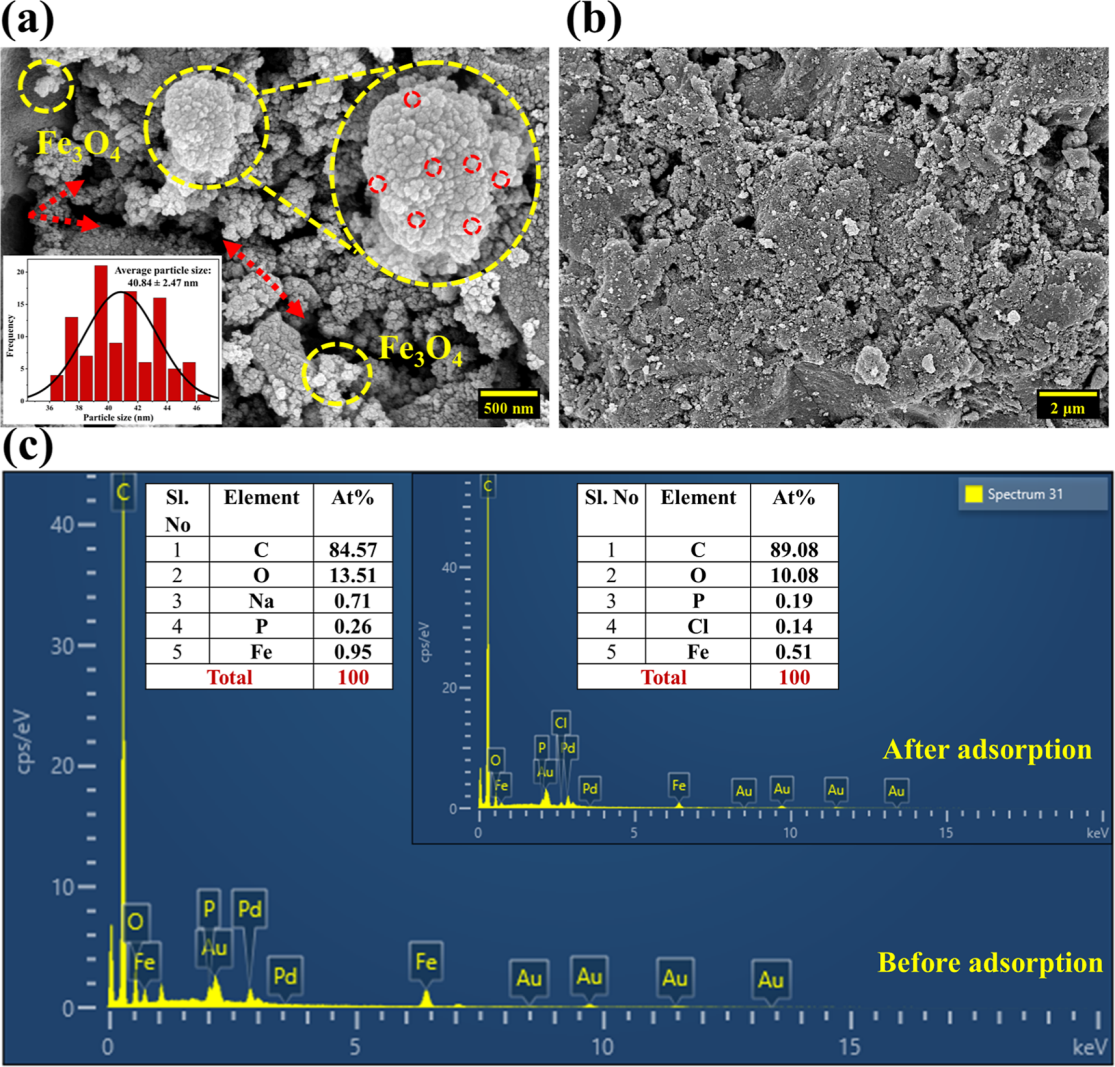
FESEM image of SCMAC (a) before and (b) after adsorption; (c) EDS spectra of SCMAC before and after adsorption.

Complementing the morphological observations, the corresponding elemental composition from energy-dispersive X-ray spectroscopy (EDS) further validates the adsorption process. Before adsorption (Figure 1c), SCMAC consisted primarily of carbon (84.57%), reflecting its carbonaceous nature, with oxygen (13.51%) indicating the existence of oxygen-rich functional moieties introduced during activation.^23^ Additionally, trace amounts of sodium, derived from sodium bicarbonate (NaHCO_3_) used for neutralization, and phosphorus, originating from H_3_PO_4_ as the activating agent, were observed. The existence of iron confirms the incorporation of iron oxide nanoparticles, imparting the desired magnetic properties to the material.^24^ After adsorption, significant changes in elemental composition were observed. The carbon content increased to 89.08%, reflecting the deposition of carbon-rich MG dye onto the SCMAC surface (Figure 1c). Simultaneously, the oxygen content decreased to 10.08%, possibly due to the interaction of MG dye molecules with the oxygen-containing functional groups on the SCMAC. A slight reduction in Fe content, from 0.95 to 0.51%, is likely a result of partial masking of Fe sites by the adsorbed dye molecules.^25^ Trace amounts of chlorine (0.14%) appeared in the postadsorption EDS, originating from the MG dye itself. Thus, the structural changes observed in the FESEM images, coupled with the compositional shifts detected in the EDS analysis, confirm the effective adsorption of MG dye onto the SCMAC.

### 2.2 Surface Area and Pore Structure of *S. campanulata*

BET surface area analysis revealed that SCMAC possesses a high specific surface area (SSA) of 1012.9 m^2^/g and a total pore volume of 0.9191 cm^3^/g. This SSA is considerably higher than those reported for MACs derived from other biomass sources, such as *Juniperus procera* leaves (38.44 m^2^/g),^26^ palm empty fruit bunch (182.2 m^2^/g),^27^ coconut shells (238–372 m^2^/g),^28^ chestnut shell (389.23 m^2^/g),^29^ peanut shells (526.34 m^2^/g),^12^ and industrial food waste (822 m^2^/g).^30^ Remarkably, it also surpasses the surface area of the AC derived from *S. campanulata* flowers synthesized in our previous study (986.41 m^2^/g).^7^ This enhancement is attributed to the incorporation of magnetic nanoparticles,^31^ which induce microstructural changes in the carbon matrix by increasing surface roughness and modifying pore structure.^32^

The nitrogen adsorption–desorption curve of SCMAC (Figure 2) exhibits a Type IV profile, characteristic of mesopores (2–50 nm),^23^ as classified by IUPAC. This is evidenced by the steady increase in nitrogen uptake across a broad relative pressure range and a well-defined hysteresis loop observed at higher pressures (*P*/*P*_0_ ≈ 0.4–1.0).^33^ Correspondingly, Barrett–Joyner–Halenda (BJH) model desorption analysis revealed an average pore diameter of 2.97 nm, firmly placing SCMAC within the mesoporous range. The pore size distribution curve (Figure 2 inset) shows a sharp and dominant peak between 2.5 and 3.5 nm, with negligible contribution beyond 10 nm, indicating a narrow and uniform pore distribution.^34^

**Figure 2:**
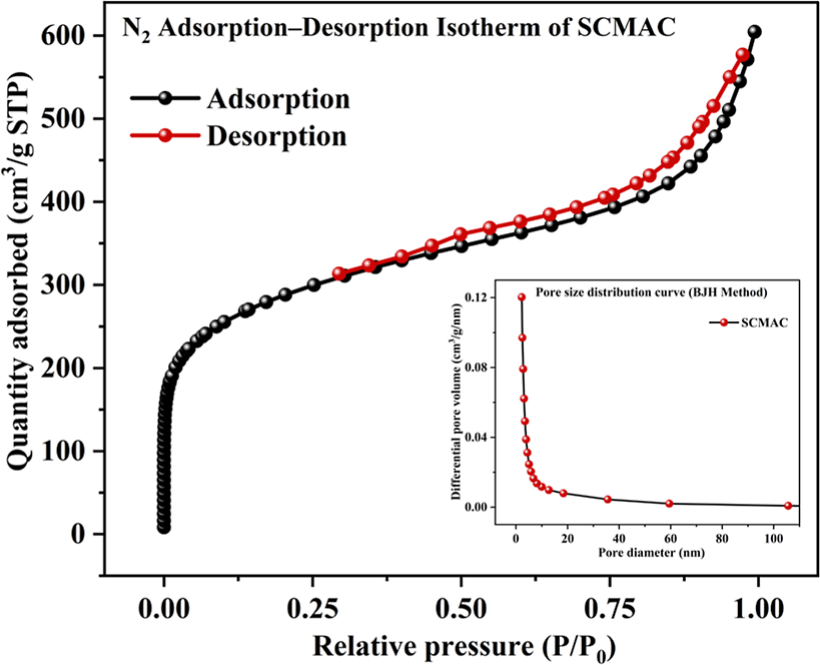
Nitrogen adsorption–desorption isotherm at liquid nitrogen temperature (77 K) of SCMAC. Inset: Barrett–Joyner–Halenda (BJH) model pore size distribution curve obtained from the desorption branch of the isotherm.

This well-developed mesoporous framework provides efficient diffusion pathways and exposes abundant adsorption sites for MG molecules. The development of this hierarchical porous structure results from the combined effect of H_3_PO_4_ activation and thermal treatment at 400 °C. Acting as both a chemical activator and a dehydrating agent, H_3_PO_4_ promotes the breakdown of cellulose and hemicellulose,^35^ preceding the decomposition and volatilization of organic components,^36^ thereby creating an intricate porous network.

### 2.3 X-ray Diffraction Analysis

A broad peak observed at 2θ = 26^ο^ corresponds to the (002) plane of graphitic structures, which is indicative of amorphous carbon (Figure S1a). This finding aligns with the results of Altintig et al., who reported a 2θ value of 21° for MAC derived from Chestnut shell.^29^ Sharper diffraction peaks at 2θ values of 30.82°, 36.07°, 43.80°, 56.34°, and 63.20°, relating to the (220), (311), (400), (422), and (440) planes, respectively.^37^ These reflections are consistent with the standard JCPDS card for crystalline Fe_3_O_4_ (JCPDS no. 19-0629) and match previously reported values for MAC derived from peanut shells.^12^ Interestingly, after MG adsorption, subtle shifts were observed in both the graphitic and Fe_3_O_4_ peaks, suggesting changes in the lattice spacing within the SCMAC structure. These shifts may result from interactions between MG molecules and the SCMAC surface, potentially causing slight expansions or contractions in the crystal lattice.^38^

### 2.4 Fourier Transform Infrared Spectroscopy Results

The FTIR spectra (Figure S1b) reveal key functional groups in SCMAC and their role in MG adsorption. The broad signal at 3896 cm^–1^ represents O–H stretching vibrations, indicating the presence of hydroxyl groups within the SCMAC structure.^39^ After MG adsorption, the slight shift of this band to 3898 cm^–1^ reflects changes in the hydrogen-bonding environment, indicating interactions between hydroxyl groups and MG molecules. The 3059 cm^–1^ signal before adsorption is assigned to C–H stretching vibration, which is typically associated with sp^2^ hybridized carbons in aromatic rings or sp^3^ hybridized carbons in aliphatic chains in SCMAC.^12^ After MG adsorption, the peak shifts to 3055 cm^–1^, indicating π–π interaction among the MG aromatic rings and the graphitic phase of SCMAC. Supporting this, 1583 cm^–1^ signal can be assigned to C=C stretching vibrations, confirming the aromatic structures within SCMAC.^12^ The slight shift of this band to 1587 cm^–1^ after adsorption further reinforces the role of π–π interactions.

The peak at 1691 cm^–1^, corresponding to C=O (carbonyl) stretching, likely results from oxidation processes during the activation of the carbon.^7^ Additionally, the peak connected with C–O stretching vibration, initially at 939 cm^–1^, shifts to 945 cm^–1^ postadsorption. This shift indicates the involvement of oxygen-rich functional groups (such as phenols or alcohols) in the adsorption process, where hydrogen bonding between these groups and MG molecules plays a significant role. Furthermore, the band at 505 cm^–1^, attributed to Fe–O vibrations in Fe_3_O_4_, confirms the successful incorporation of Fe_3_O_4_ nanoparticles into SCMAC.^40^ Postadsorption, this band shifts slightly to 509 cm^–1^, likely due to weak interactions with MG molecules, potentially involving surface complexation or ligand exchange between the dye’s functional moieties and Fe^3+^/Fe^2+^ ions on the adsorbent surface. Thus, the shifts observed in the FTIR peaks after adsorption provide clear evidence of strong interaction among the MG molecules and the SCMAC surface.

### 2.5 X-ray Photoelectron Spectroscopy Studies

The XPS analysis provides detailed insights into the surface chemistry of SCMAC before and after MG dye adsorption. The full-survey spectrum (Figure 3a) prior adsorption shows prominent peaks for C 1s (71.65%), O 1s (12.4%), and Fe 2p (2.47%). The high carbon content reflects the carbonaceous nature of the AC matrix, while the oxygen content is owing to hydroxyl and carboxyl groups formed during the activation process. The presence of iron further validates the successful incorporation of iron oxides, responsible for the magnetic nature. After adsorption, notable changes were observed in the elemental composition of the SCMAC surface. The decrease in carbon quantity to 68.1% could be because of the coverage of the SCMAC surface by MG dye molecules, reducing the detectable carbon signal from the underlying matrix. Concurrently, the oxygen content increased to 14.46%, indicating the reorganization or involvement of surface functional groups in interactions with MG molecules. Furthermore, the iron content (Fe 2p) increased slightly to 2.55%, suggesting a greater surface exposure of iron oxide species, possibly due to surface rearrangements during adsorption. These compositional changes in carbon, oxygen, and iron after MG adsorption are consistent with trends reported in previous studies.^41,42^

**Figure 3:**
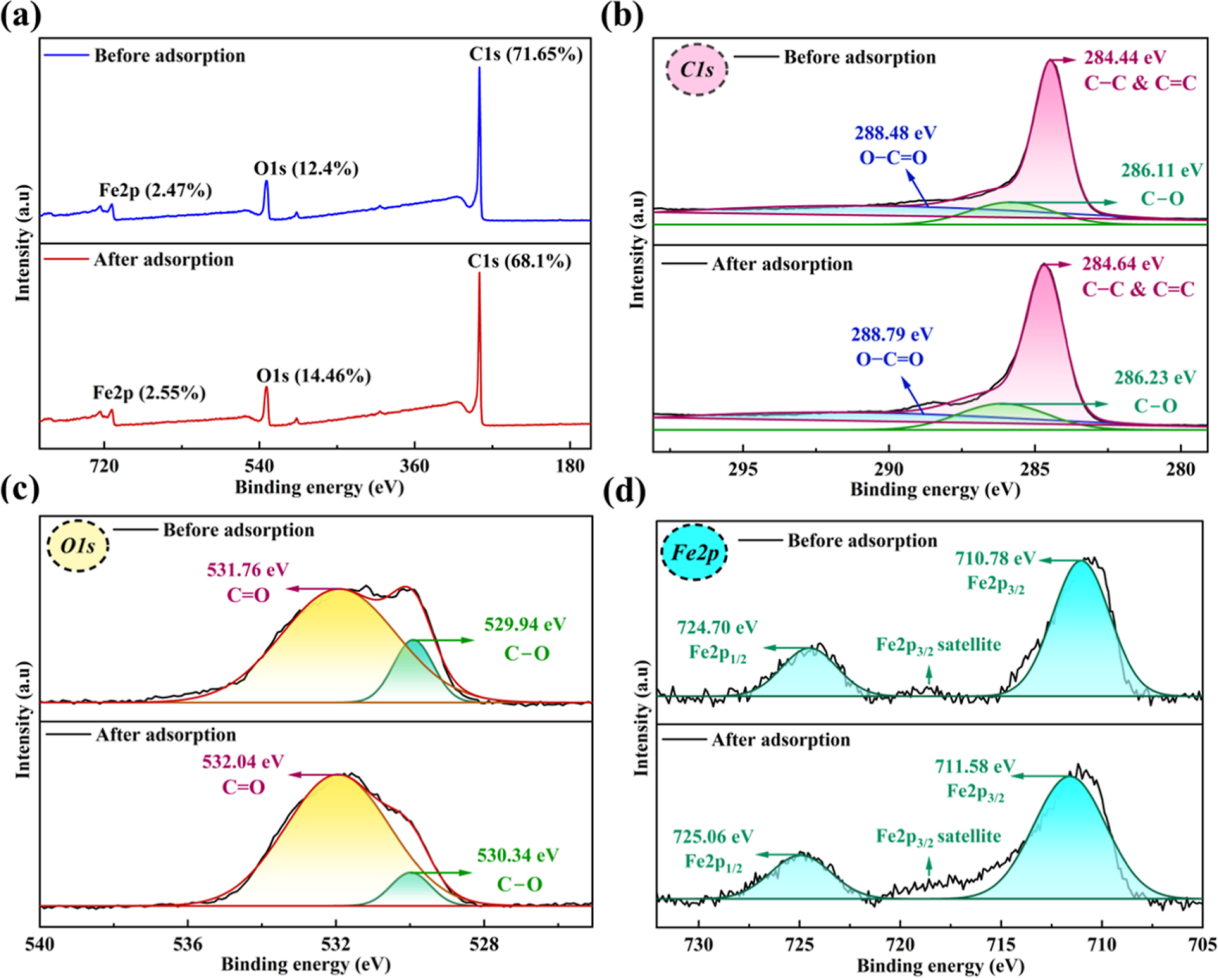
XPS spectra of SCMAC before and after adsorption showing (a) full survey spectrum (b) C 1s, (c) O 1s and (d) Fe 2p spectra.

Before MG adsorption, high-resolution deconvolution of the C 1s region (Figure 3b) reveals three main parts: The dominant signal at 284.44 eV corresponding to graphitic or aliphatic C–C/C=C bonds, a shoulder at 286.11 eV attributed to C–O functionalities, and a less intense component at 288.48 eV associated with carboxyl or ester groups (O=C–O).^42^ Postadsorption, the principal C–C/C=C peak shifted to 284.64 eV, suggesting alterations in the electronic environment of the carbon atoms, potentially due to π–π interactions or surface coverage by MG molecules, which can obscure or attenuate the underlying graphitic signals.^12,25^ The C–O peak also shifted slightly to 286.23 eV, reflecting interactions between MG and oxygen-rich functional groups on the SCMAC surface. Similarly, the O=C–O peak shifted to 288.79 eV, supporting the involvement of these groups in the adsorption process, likely through hydrogen bonding interactions similar to those influencing the C–O shift.

Before adsorption, the O 1s spectrum (Figure 3c) exhibits two main peaks: one at 531.76 eV (C=O) and the other at 529.94 eV (C–O) in SCMAC. After MG adsorption, the C=O peak shifted to 532.04 eV, suggesting interaction between MG dye molecules and the carbonyl moieties of SCMAC.^25^ Similarly, the C–O peak shifted to 530.34 eV, indicating possible hydrogen bonding between the dye and oxygen atoms in the C–O functional groups.

Before MG adsorption, the Fe 2p spectrum (Figure 3d) exhibited two prominent peaks characteristic of Fe_3_O_4_ at binding energies of 710.78 eV (Fe 2p 3/2) and 724.70 eV (Fe 2p 1/2). Additionally, a satellite peak corresponding to Fe 2p 3/2 was observed, further confirming the presence of Fe_3_O_4_ on the SCMAC surface. These spectral positions are consistent with the mixed-valent oxidation state of Fe_3_O_4_, which contains both Fe^2+^ and Fe^3+^ species.^43^ After MG adsorption, both Fe 2p peaks shifted slightly, with the Fe 2p 3/2 peak moving to 711.58 eV and the Fe 2p 1/2 peak shifting to 725.06 eV. These subtle changes in binding energy indicate minor alterations in the chemical state or local coordination environment of iron atoms, likely resulting from partial complexation with functional groups present in the MG dye.^44^ These XPS findings, when considered alongside the observed FTIR shifts, provide robust evidence of strong interfacial interactions between MG and both the oxygen-containing functional groups and Fe_3_O_4_ species on SCMAC.

### 2.6 Vibrating Sample Magnetometer Analysis

The hysteresis loops of SCMAC provide valuable insights into its magnetic behavior. The initially narrow loop, characteristic of superparamagnetism, becomes even narrower postadsorption, confirming the retention of this property for effective magnetic separation (Figure 4). Before adsorption, the saturation magnetization (*M*_s_) and the retentivity (*M*_r_) were 1.39 and 0.023 emu/g respectively (Figure 4 inset), resulting in a *M*_r_/*M*_s_ ratio of 0.0165, which remains well below 0.25, indicating strong superparamagnetic behavior.^45^ After MG adsorption, both *M*_s_ and *M*_r_ decreased to 0.62 and 0.011 emu/g, respectively (Figure 4 inset), leading to a slightly higher *M*_r_/*M*_s_ ratio of 0.018, which again remains well below 0.25, confirming that the adsorption process does not significantly affect the superparamagnetic nature of SCMAC.^45^ Such declines in magnetic properties are commonly observed in magnetic carbon materials and are typically attributed to surface fouling by adsorbed dye molecules,^20^ partial detachment or redistribution of magnetic nanoparticles,^42^ and diminished magnetic domain alignment resulting from surface modifications during adsorption. Similar observation in *M*_s_ have been reported in the literature for magnetic biochar from cotton stalks (*M*_s_: 13.96–4.34 emu/g, 68.9% reduction)^46^ and MAC derived from the oak pericarp, (*M*_s_: 3.77–1.71 emu/g, 54.6% reduction),^45^ where such changes did not hinder magnetic separability.

**Figure 4:**
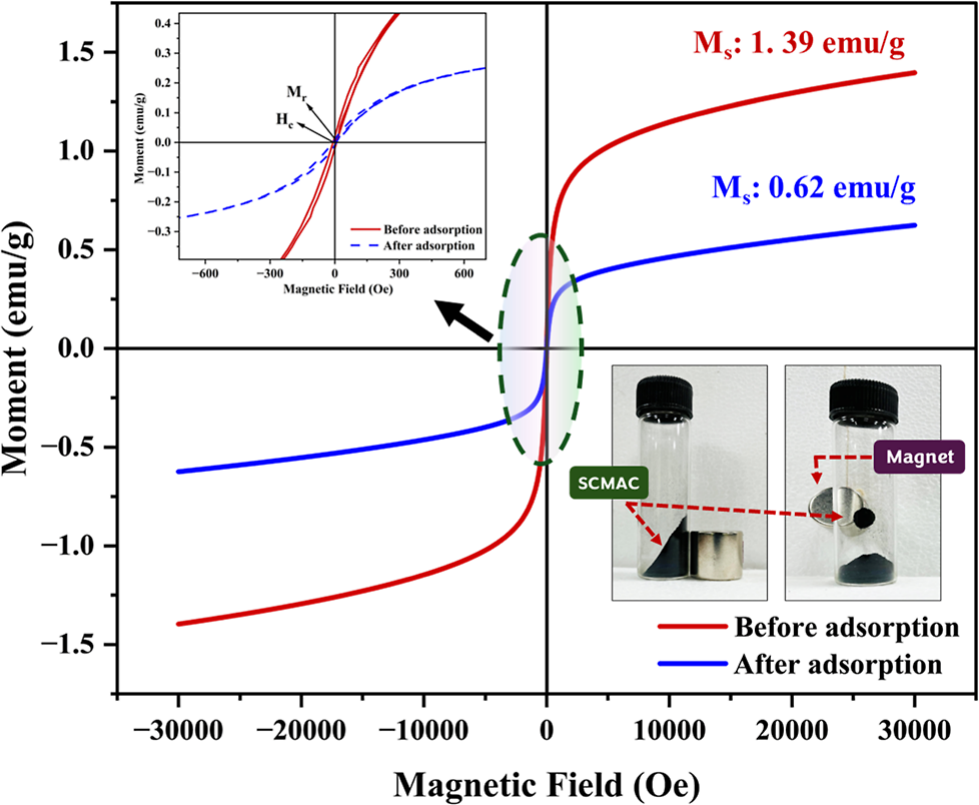
Magnetization curves of SCMAC at 300 K before and after adsorption.

A slight increase in coercivity (*H*_c_) from 9.58 to 10.23 Oe (Figure 4) postadsorption suggests minor alterations in the surface properties and magnetic domain structure of the adsorbent material.^25^ The observed magnetic alterations in the current study are consistent with XRD results (Figure S1a), where subtle peak broadening and intensity reduction postadsorption indicate a decrease in crystallinity, likely caused by surface coverage of SCMAC by MG dye molecules.

### 2.7 Influence of pH

At pH 3, which is below the point-of-zero charge (PZC) of SCMAC (3.5) (Figure S2), the surface of SCMAC is positively charged. Simultaneously, MG, with a p*K*_a_ of 6.9, exists predominantly in its cationic form,^47^ which results in electrostatic repulsion, leading to a relatively low adsorption efficiency (%*R*) of 63.25% (Figure 5a). As the pH increases from 3 to 6, the surface charge of SCMAC becomes progressively less positive and eventually turns negative as the pH moves above the PZC. In this range, MG remains largely in its cationic form due to the presence of a quaternary ammonium nitrogen group (−N^+^(CH_3_)_2_). The reduction in electrostatic repulsion among the SCMAC and MG facilitated a dramatic increase in adsorption (Figure 5a). The adsorption efficiency peaked at pH 6, reaching 99.37%, where the electrostatic interactions among the negatively charged SCMAC surface and the positively charged MG molecules were highly favorable. A similar trend of low removal efficiency at pH 3, followed by an increase as the pH rises, was also reported for MG adsorption using AC derived from spent tea leaves.^45^ At pH levels beyond 6, the adsorption efficiency showed a slight decline. As the pH approached the p*K*_a_ of MG, a small fraction of the dye began to transition into its neutral form, reducing the proportion of cationic MG available for adsorption. Additionally, at higher pH values, competition from OH^–^ ions for adsorption spots on the SCMAC surface may have contributed to this decrease.^48^ Due to the minimal variation in adsorption performance between pH 4 and 6 (96.41% and 99.37%, respectively), pH 4-corresponding to the native pH of the MG solution, was selected for further studies to eliminate the need for pH adjustment and maintain operational simplicity. The consistent MG removal within this pH range is also reported in a study on AC derived from gasified *Hevea brasiliensis* root.^49^

**Figure 5:**
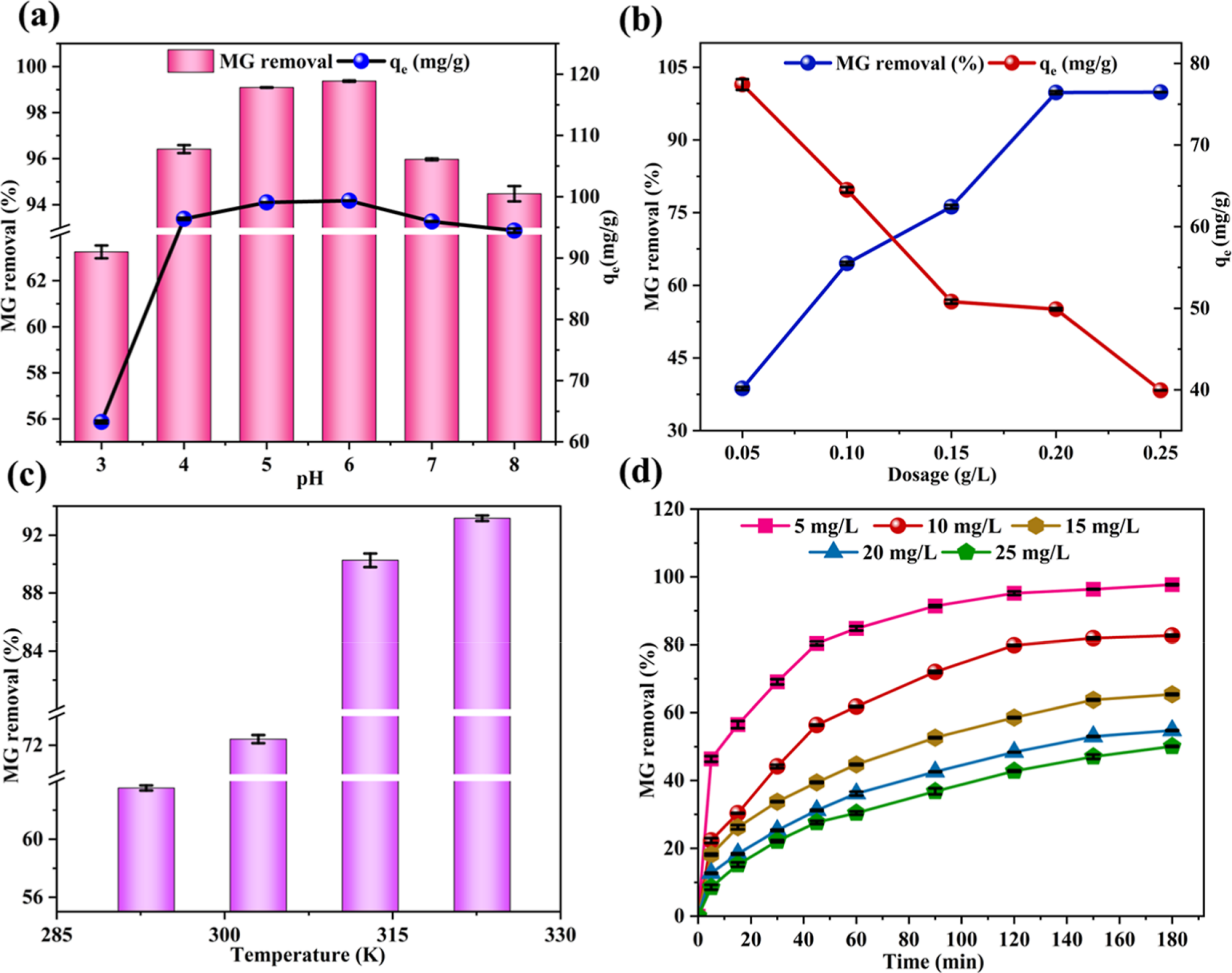
Adsorption parameters including (a) influence of pH, (b) effect of dosage, (c) effect of temperature and (d) influence of initial MG concentration and contact time for the adsorption of MG onto SCMAC.

### 2.8 Influence of Dosage

The adsorption performance of SCMAC exhibited a clear dosage-dependent behavior, influencing both %*R* and *q*_e_. At the lowest SCMAC dosage (0.05 g/L), MG removal efficiency was relatively low (38.70%), likely due to an insufficient number of active adsorption spots (Figure 5b). However, the equilibrium adsorption capacity (*q*_e_, mg/g) was highest at this dosage, indicating efficient utilization of the limited active sites. As the SCMAC dosage increased, MG removal efficiency improved significantly, reaching near-complete removal (99.86%) at the highest dose of 0.25 g/L (Figure 5b). Conversely, *q*_e_ decreased with increasing dosage, which can be attributed to factors such as abundance of unsaturated adsorption sites, reduced concentration gradient of MG dye between the bulk solution and the adsorbent, and potential aggregation of SCMAC particles, which may reduce the effective surface area for adsorption.^50,51^ This inverse relationship between adsorbent dosage and *q*_e_ is consistent with previous studies using AC prepared from raw corncob^51^ and mesoporous magnetic corn straw-derived biochar.^25^ According to the investigation, the optimal dosage of SCMAC was identified as 0.15 g/L, achieving a removal efficiency of 76.23% and a *q*_e_ of 50.82 mg/g.

### 2.9 Influence of Temperature

The data reveals a clear positive trend in the adsorption efficiency of MG by SCMAC with increasing temperature (Figure 5c). As the temperature rises from 293 to 323 K, the %R increases significantly, improving from 63.53 to 93.16%. This trend highlights the endothermic nature of the adsorption process, where higher temperatures enhance the mobility of MG molecules, reduce solution viscosity, and promote greater interaction with active sites^52^ on SCMAC. Similar behavior has been reported in studies utilizing AC derived from coffee waste^53^ and chestnut shells.^29^ Although elevated temperatures improve adsorption performance, operating at a higher temperature of 323 K may lead to higher energy demands, thereby reducing cost-efficiency and practicality for large-scale applications. To balance performance with feasibility, an optimal temperature of 303 K was chosen. This operating temperature aligns with similar studies on MG adsorption using AC prepared from jackfruit^54^ and modified *Luffa aegyptica* peel.^55^

### 2.10 Influence of Initial Dye Concentrations and Contact Duration

The %*R* of MG consistently increased with contact time across all initial concentrations, as shown by the characteristic adsorption curves (Figure 5d). In the initial stages, the adsorption rate was rapid owing to the abundance of active sites on SCMAC, allowing efficient binding of MG molecules. This phase, typically within the first 30 min, demonstrated a significant increase in removal efficiency. As the active sites became progressively saturated, the adsorption rate slowed, and the process entered a more gradual phase between 60 and 90 min^11^ By 120 min, the system approached equilibrium, with minimal changes in %*R*, primarily due to diffusion limitations and repulsive interactions between MG molecules in the bulk and adsorbed phase, which hinder further occupation of active sites on the SCMAC surface.^56^ Additionally, the initial concentration of MG strongly influenced the adsorption process. Lower concentrations exhibited higher removal efficiencies due to the favorable ratio of active sites to MG moieties, while higher concentrations led to reduced %*R* as the limited active sites were rapidly saturated.^24^

### 2.11 Adsorption Kinetics

Among the adsorption models studied, the pseudo-second order (PSO) model emerged as the most effective in describing the adsorption of MG onto SCMAC (Figure 6a). This model identifies chemisorption, involving the formation of chemical bonds between MG molecules and the SCMAC surface, as the rate-limiting step. The close agreement between the experimentally defined equilibrium adsorption capacity (*q*_e,exp_) of 32.55 mg/g and the calculated capacity (*q*_e,cal_) of 33.21 mg/g (Table 1) underscores the reliability of this model. Statistical validation further supports this conclusion, with a high *R* ^2^ value of 0.9720 and a reduced chi-square (χ^2^) value of 3.29, indicating a superior fit to the experimental data set. These findings align with other studies that have demonstrated the suitability of the PSO model for MG adsorption using MAC derived from *Fraxinus chinensis*,^57^ sugar cane bagasse,^11^ and *Juniperus procera* leaves.^26^

**Figure 6:**
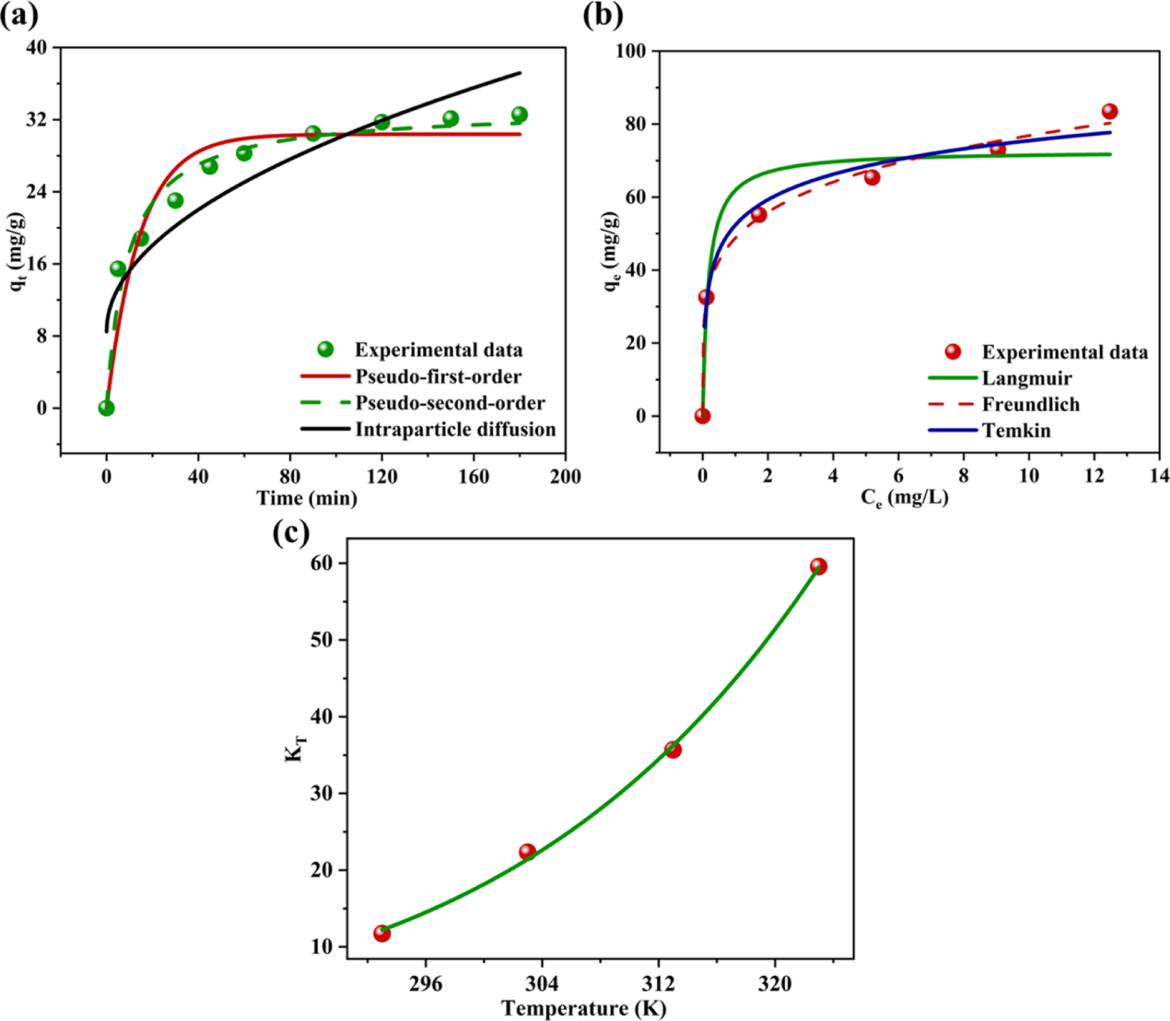
(a) Adsorption kinetics, (b) adsorption isotherm and (c) Van’t Hoff plot for the adsorption of MG onto SCMAC.

**Table 1:** Parameters of Kinetic, Isotherm, and Thermodynamic Models for the Adsorption of MG Onto SCMAC^a^

kinetic models		
kinetic model	equation	parameters
pseudo-first-order (PFO)	*q*t = *q*e(1 – exp(−*k*_1_ *t*))	*k*_1_ (min^–1^) *=* 0.0689
		*q*_e_,_cal_ (mg/g) *=* 30.39
		*R*^2^ = 0.9219
		χ^2^ = 9.21
pseudo-second-order (PSO)	$q_{t}=\frac{q_{e}^{2}k_{2}t}{{q_{e}k}_{2}t+1}$	*k*_2_ (g/mg min) *=* 0.0032
		*q*_e_,_cal_ (mg/g) = 33.21
		*R*^2^ = 0.9720
		χ^2^ = 3.29
intraparticle diffusion (IPD)	*q*_t_ = *K*_diff_ *t* ^0.5^ + *C*	*K*_diff_ ((mg/g) min^0.5^) = 2.137
		*C* (mg/g) = 8.49
		*R*^2^ = 0.8454
		χ^2^ = 18.22

isotherm models		
isotherm model	equation	parameters
Langmuir	$q_{e}=\frac{q_{m}K_{L}C_{e}}{\left({1+K_{L}C}_{e}\right)}$	*q*_m_ (mg/g) *=* 72.73
		*K*_L_ (L/mg) *=* 5.68
		*R*^2^ *=* 0.937
		χ^2^ = 74.37
Freundlich	*q*_e_ = *K*_F_ *C*_e_^1/*n*^	*K*_F_ ((mg/g)/(mg/L)^1/*n*^) = 48.78
		1/*n* = 0.197
		*R*^2^ = 0.995
		χ^2^ = 5.38
Temkin	*q*_e_ = *B*_T_ ln(*K*_t_ *C*_e_)	*B*_T_ (J/mol) = 10.06
		*K*_t_ (L/g) = 181.71
		*R*^2^ = 0.960
		χ^2^ = 19.62

thermodynamic model				
*T* (K)	293	303	313	323
Δ*G*° (kJ/mol)	–5.99	–7.72	–9.39	–11.04

equation	parameters
$K_{T}=\exp\left[\left(\frac{\Delta S^{{}^{\circ}}}{R}\right)-\left(\frac{\Delta H^{{}^{\circ}}}{R}\right)\frac{1}{T}\right]$	Δ*H*° (kJ/mol) = 41.52
	Δ*S*° (J/mol K) = 162.51
	*R*^2^ = 0.999
	χ^2^ = 0.701

a *q*_e_: equilibrium adsorption capacity; *k*_1_: PFO constant; *k*_2_: PSO constant; *K*_diff_: IPD rate constant; *C*: IPD intercept; *q*_m_: monolayer adsorption capacity; *K*_L_: Langmuir constant; *K*_F_: Freundlich constant; 1/*n*: adsorption intensity; *C*_e_: equilibrium MG concentration; Δ*G*°: (=–*RT* ln *K*_T_), standard Gibbs free energy; *K*_T_: (*q*_e_/*C*_e_), distribution factor; Δ*H*°: standard enthalpy change and Δ*S*°: standard entropy change; *B*_T_ & *K*_t_: Temkin constants.

In contrast, the pseudo-first order (PFO) model, which typically represents systems dominated by weaker physical interactions, was less effective in capturing the adsorption kinetics of MG onto SCMAC (Figure 6a). Although the PFO model showed a reasonable *R* ^2^ value of 0.9219, its higher χ^2^ value of 9.21 (Table 1) indicates a poorer fit compared to the PSO model. Intraparticle diffusion (IPD) model was also employed to explore possible diffusion limitations. The nonlinear IPD plot (Figure 6a) exhibited a nonzero intercept (*C* = 8.49 mg/g) (Table 1), indicating that IPD contributes to the overall rate but is not the sole rate-limiting step. The relatively lower *R* ^2^ value of 0.8454 and higher χ^2^ of 18.22 further support this interpretation. These observations suggest that external mass transfer and boundary layer effects also influence the adsorption process, particularly during the initial stages.^58^

However, it is important to note that the fitting of the PSO model alone does not definitively confirm chemisorption as the sole adsorption mechanism. Additional spectroscopic analyses namely FTIR and XPS provide supporting evidence. The observed shifts in key functional groups such as O–H, C=O, C–O, and Fe–O along with changes in binding energies of C 1s, O 1s, and Fe 2p signals, confirm strong interfacial interactions between MG molecules and SCMAC. These include hydrogen bonding, π–π interactions, and surface complexation involving both oxygenated functional groups and Fe^2+^/Fe^3+^ sites on the Fe_3_O_4_ surface. Therefore, although multiple steps such as external diffusion and IPD contribute to the overall adsorption, the combined kinetic and spectroscopic evidence strongly supports chemisorption as the predominant mechanism governing MG uptake by SCMAC.

### 2.12 Adsorption Isotherm

The Langmuir model predicted a high maximum monolayer adsorption capacity (*q*_m_) of 72.73 mg/g (Table 1), indicating the potential of SCMAC to adsorb significant amounts of MG under saturating conditions. The dimensionless separation factor (*R*_L_) was calculated as 0.034 (0 < *R*_L_ < 1) using an initial MG concentration (*C*_i_) of 5 mg/L, confirming the favorable nature of the adsorption process. Although the Langmuir model yielded a reasonable *R* ^2^ value of 0.937, the relatively high χ^2^ of 74.37 suggests some deviations from the ideal assumptions of monolayer adsorption on a homogeneous surface (Figure 6b). Nonetheless, the *q*_m_ value obtained in this study surpasses many previously reported adsorption capacities for MG using AC-based adsorbents, as summarized in Table S1.

In contrast, the Freundlich isotherm provided a superior fit (Figure 6b), with the highest *R* ^2^ value of 0.995 and a significantly lower χ^2^ value of 5.38 (Table 1). The heterogeneity factor, ‘*n*’ (=5.07) and corresponding 1/*n* value of 0.197 reflect strong adsorption intensity and pronounced surface heterogeneity, especially at lower MG concentrations. This model suggests multilayer adsorption on an energetically diverse surface, which aligns well with the structural complexity of SCMAC. A similar preference for the Freundlich model was reported in studies using AC derived from coconut shells^59^ and *Catha edulis* stem.^48^

The Temkin isotherm, which accounts for adsorbate–adsorbent interactions by considering the effect of adsorption energy, also described the system with moderate accuracy (*R* ^2^ = 0.960, χ^2^ = 19.62) as described in Table 1. However, it was less precise than the Freundlich model (Figure 6b), further confirming that adsorption of MG onto SCMAC occurs predominantly on a heterogeneous surface through multilayer interactions.

### 2.13 Thermodynamic Analysis

The nonlinear van’t Hoff plot (*R* ^2^ = 0.999) confirmed the consistency and reliability of the temperature-dependent equilibrium data (Figure 6c). Precisely, the increasing magnitude of calculated Gibbs free energy (Δ*G*°) with rising temperature (Table 1), indicates enhanced spontaneity, a behavior consistent with previous studies on MG adsorption using banana peel—based AC^60^ and cotton stalk biochar.^61^ This trend is accompanied by a positive entropy change (Δ*S*° = 162.51 J/mol K), which signifies increased randomness at the solid–liquid interface, likely resulting from desolvation and structural reorganization^62^ of MG dye molecules during adsorption. The enthalpy change (Δ*H*°) was found to be 41.52 kJ/mol, indicating an endothermic adsorption process. While enthalpy values above 40 kJ/mol are generally indicative of chemisorption^63^ such magnitudes can also result from strong physisorption mechanisms. To strengthen this interpretation, thermodynamic results were correlated with kinetic modeling, where the PSO model provided the best fit. This was further supported by FTIR and XPS analyses, which revealed distinct shifts in key functional groups, along with evidence of π–π interactions and surface complexation. Consequently, while physisorption may contribute during the early stages of adsorption, the combined thermodynamic, kinetic, and spectroscopic evidence clearly indicates that chemisorption is the dominant pathway for MG removal by SCMAC.

### 2.14 Spiking Studies

Spiking studies were performed to evaluate the adsorption performance of SCMAC in different water matrices, thereby assessing its real-world applicability. As illustrated in Figure 7a, SCMAC achieved the highest %*R* in distilled water (DW) or control (CO) sample, reaching 84.73%. In real water samples, %*R* ranged from 77.02% in Yamuna (YA) to 82.54% in both Sai (SI) and industrial ground water (IGW). The slight reduction in adsorption efficiency in these complex matrices, compared to DW, is attributed to the presence of coexisting ions such as chlorides, bicarbonates, sulfates, and dissolved organic matter,^7^ which can compete with MG molecules for active adsorption sites. Despite these competitive interferences, SCMAC demonstrated stable and robust adsorption behavior across all tested conditions. In IGW samples, UV–visible spectral analysis further confirmed the efficacy of SCMAC, evidenced by a pronounced reduction in MG absorbance intensity after treatment (Figure 7b). These results collectively highlight the adaptability of SCMAC for practical applications in varied water environments, reinforcing its promise as a sustainable and cost-effective adsorbent for environmental remediation and dye-contaminated wastewater treatment.

**Figure 7:**
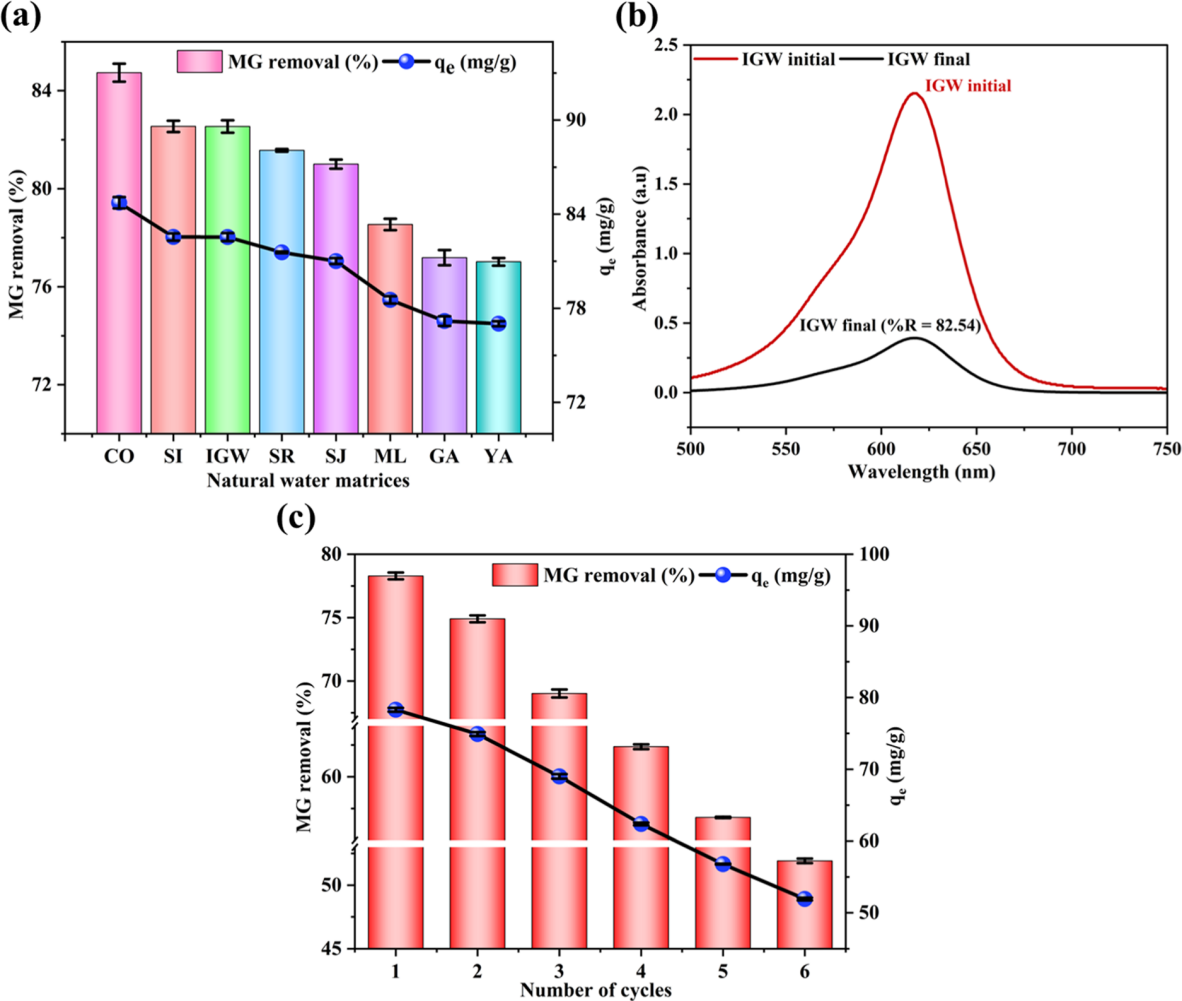
(a) MG adsorption performance using SCMAC across different water matrices; (b) UV–visible spectra of MG removal performance in industrial groundwater (IGW); (c) regeneration potential of SCMAC using methanol.

### 2.15 Desorption Studies

The reusability of SCMAC was evaluated over six consecutive adsorption–desorption cycles using methanol as the desorbing agent. Due to its polar nature and low molecular weight, methanol effectively disrupts noncovalent interactions such as hydrogen bonding, π–π stacking, and electrostatic attractions between MG molecules and the SCMAC surface.^24^ As a result, SCMAC showed high desorption efficiency in the first cycle, reaching 78.29% (Figure 7c). However, a gradual decline in performance was observed with successive cycles, with desorption efficiency reducing to 51.92% in the sixth cycle. This reduction is attributed to the partial loss of active sites, residual dye entrapment, and minor structural or chemical modifications on the adsorbent surface.^64^ Despite this, SCMAC retained 66.3% of its initial removal efficiency, demonstrating reliable reusability and operational stability.

Although magnetic characterization after six regeneration cycles was not feasible due to resource constraints, previous VSM analysis confirmed the material’s superparamagnetic behavior and magnetic recoverability postadsorption (Figure 4). This assessment is essential, as the gradual loss of magnetism could hinder recoverability and raise concerns about secondary contamination due to iron leaching. However, even if magnetic properties decline over successive reuse cycles, the risk of iron release into treated water is expected to be minimal. This is supported by comparative studies wherein MAC materials with substantially higher initial *M*_s_ values (9.0 emu/g) released only 5–10 μg/L of Fe over multiple cycles, which is well below the World Health Organization (WHO) threshold of 0.3 mg/L for drinking water.^42^ Given SCMAC’s lower initial magnetization (1.39 emu/g), any potential Fe release is expected to remain far below regulatory limits. These results collectively confirm that SCMAC is not only effective in dye removal but also environmentally safe, reinforcing its potential as a promising and reusable adsorbent capable of sustaining multiple regeneration cycles under mild desorption conditions.

### 2.16 Phytotoxicity Studies and Statistical Analysis

The phytotoxicity assay was performed to evaluate the influence of MG on *Solanum lycopersicum* seeds and to evaluate the efficacy of adsorbent-treated MG solutions in mitigating toxicity. Key plant growth indicators such as germination index (GI, %), shoot length (SL, cm), and root length (RL, cm) were measured under exposure to untreated and SCMAC-treated MG solutions (5, 15, and 25 mg/L), with distilled water serving as the positive control^65–67^ (Figure 8).

**Figure 8:**
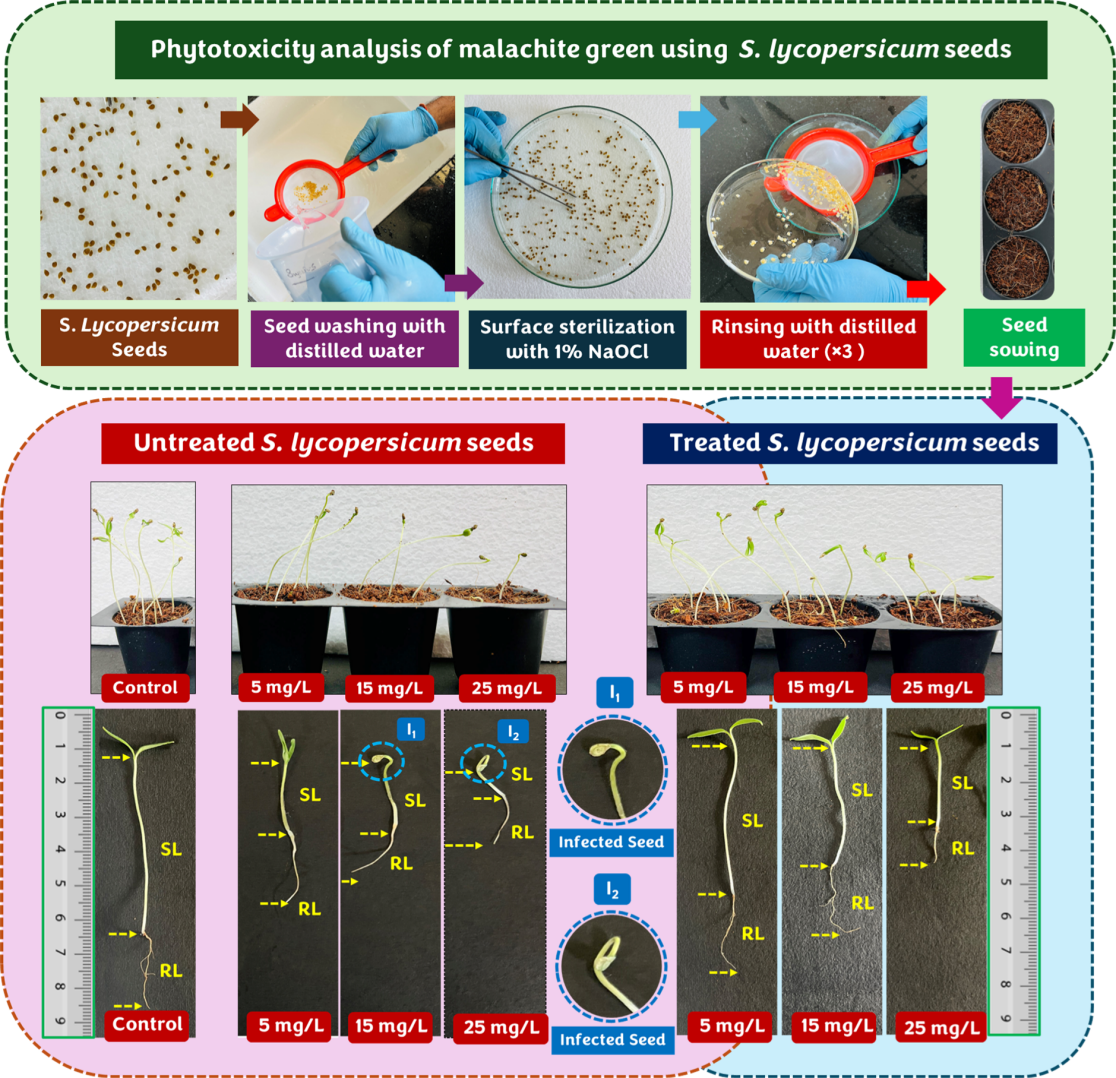
Phytotoxicity assessment of MG before and after treatment with SCMAC using *S. lycopersicum* seeds.

Untreated MG solutions caused a concentration-dependent decline in GI compared to the control (100%), decreasing significantly to 57.14% at 5 mg/L (*p* < 0.001), 42.85% at 15 mg/L (*p* < 0.001), and 28.57% at 25 mg/L (*p* < 0.001), thereby confirming the phytotoxic nature of MG (Figure 9). Visible signs of phytotoxicity, curled growth, and stunted foliage were also observed at higher MG concentrations (Figure 8 inset I1 and I2). SCMAC treatment significantly improved GI across all concentrations, with the most notable recovery observed at 15 mg/L, where GI increased from 42.85 to 71.42% (*p* < 0.001), indicating substantial detoxification (Figure 9).

**Figure 9:**
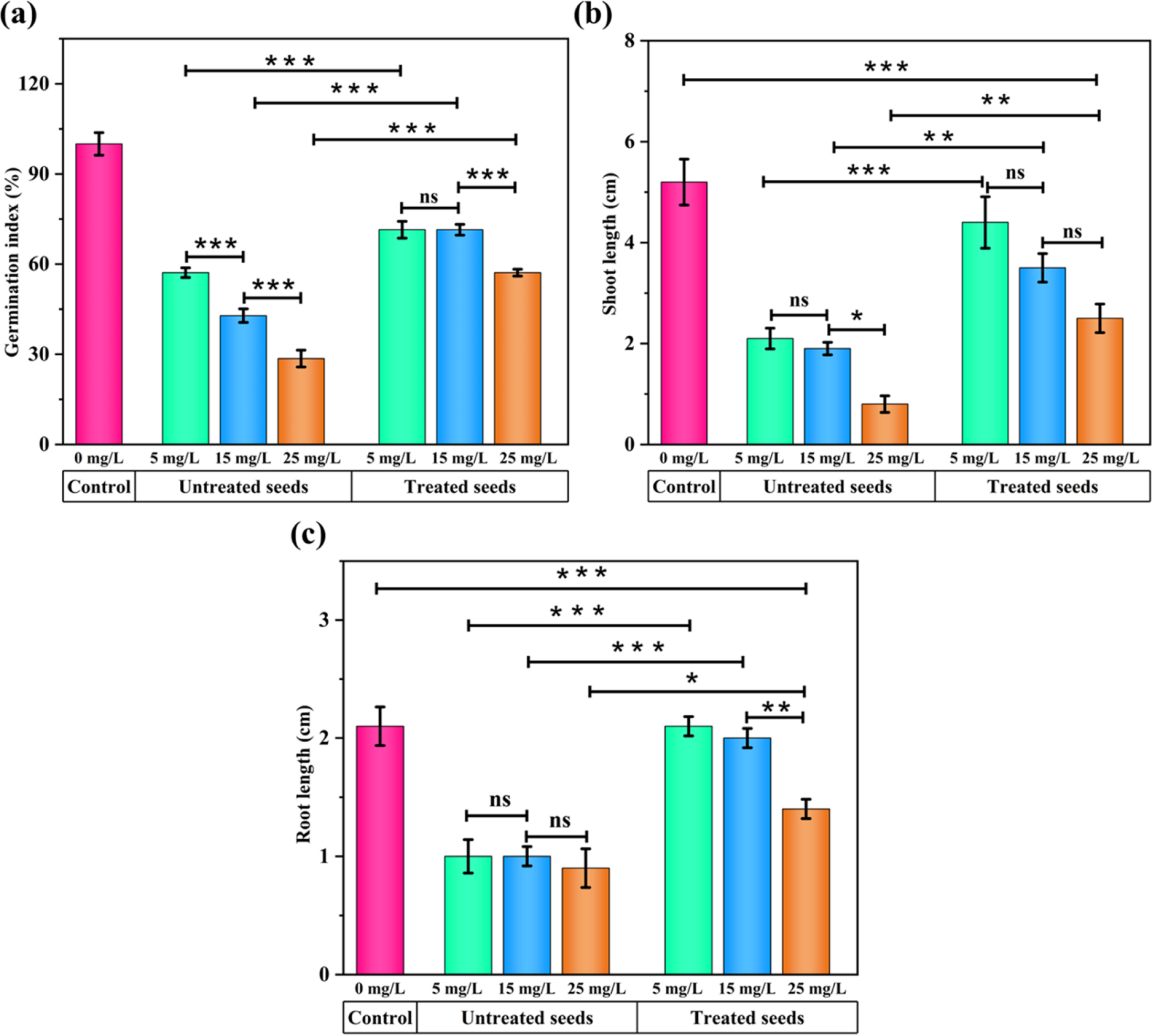
Comparative phytotoxicity analysis of untreated and SCMAC-treated MG dye solutions on *S. lycopersicum* seeds at concentrations of 5, 15, and 25 mg/L; (a) germination index (GI, %), (b) shoot length (SL, cm), (c) root length (RL, cm). Data are expressed as mean ± standard deviation (*n* = 3). Statistical analysis was performed using one-way ANOVA followed by Tukey’s HSD posthoc test (*p < 0.05, **p < 0.01, ***p < 0.001).

Shoot and root growth were also markedly inhibited by MG exposure. At 25 mg/L, SL and RL declined to 0.8 (*p* < 0.001) and 0.9 cm (*p* < 0.001) respectively, compared to 5.2 and 2.1 cm in the control group, confirming significant growth suppression (Figure 9). SCMAC treatment alleviated this toxicity, restoring SL and RL to 2.5 cm (*p* < 0.01) and 1.4 cm (*p* < 0.05), respectively, reflecting partial but statistically significant recovery. Notably, complete or near-complete recovery of both parameters was observed at lower concentrations. At 5 mg/L, SL and RL recovered to 4.4 and 2.1 cm, respectively, with no significant difference (*p* > 0.05 for both) from the control group (5.2 and 2.1 cm) (Figure 9). At 15 mg/L, SL recovered to 3.5 cm, showing a significant improvement (*p* < 0.01) over the untreated group (1.9 cm), although it remained significantly lower (*p* < 0.01) than the control (5.2 cm). Similarly, RL increased to 2.0 cm, showing significant improvement (*p* < 0.001) compared to the untreated group (1.0 cm), and remained statistically indistinguishable (*p* > 0.05) to the control (Figure 9). These findings align with a study on *Vigna mungo* seeds exposed to marmelos-derived activated biochar-treated MG dye solutions at concentrations ranging from 25 to 150 mg/L.^68^ Similarly, a study using sugar cane bagasse (SCB)-derived biochar showed complete mitigation of MG dye toxicity, with seed germination rates reaching 100%.^69^ Therefore, the findings underscore the detrimental effects of untreated MG dye on plant growth^70^ while demonstrating the efficacy of SCMAC-treated solutions in alleviating phytotoxic stress, thereby enhancing the prospects of using adsorption treatment in environmental remediation.

### 2.17 Insights on the Adsorption Mechanism

The adsorption mechanism of MG onto SCMAC (Figure 10) is governed by a combination of structural, chemical, and energetic factors, as evidenced by comprehensive characterization and adsorption studies. FESEM images revealed a highly porous morphology with embedded Fe_3_O_4_ nanoparticles (∼40.84 ± 2.47 nm), which facilitated extensive surface accessibility and magnetic separability. Postadsorption images showed pore blockage and surface smoothing, indicating substantial MG deposition. These morphological changes were corroborated by EDS analysis, where the carbon content increased from 84.57 to 89.08%, oxygen decreased from 13.51 to 10.08%, and iron reduced from 0.95 to 0.51%; in addition, chlorine appeared postadsorption, confirming the presence of the MG molecule and its interaction with SCMAC’s surface functional groups. Complementing these observations, BET analysis further supported the mesoporous structure of SCMAC, with a specific surface area of 1012.9 m^2^/g, mean pore size of 2.97 nm, and pore volume of 0.9191 cm^3^/g—parameters that provided a large and interconnected network of active sites suitable for accommodating bulky MG molecules.

**Figure 10:**
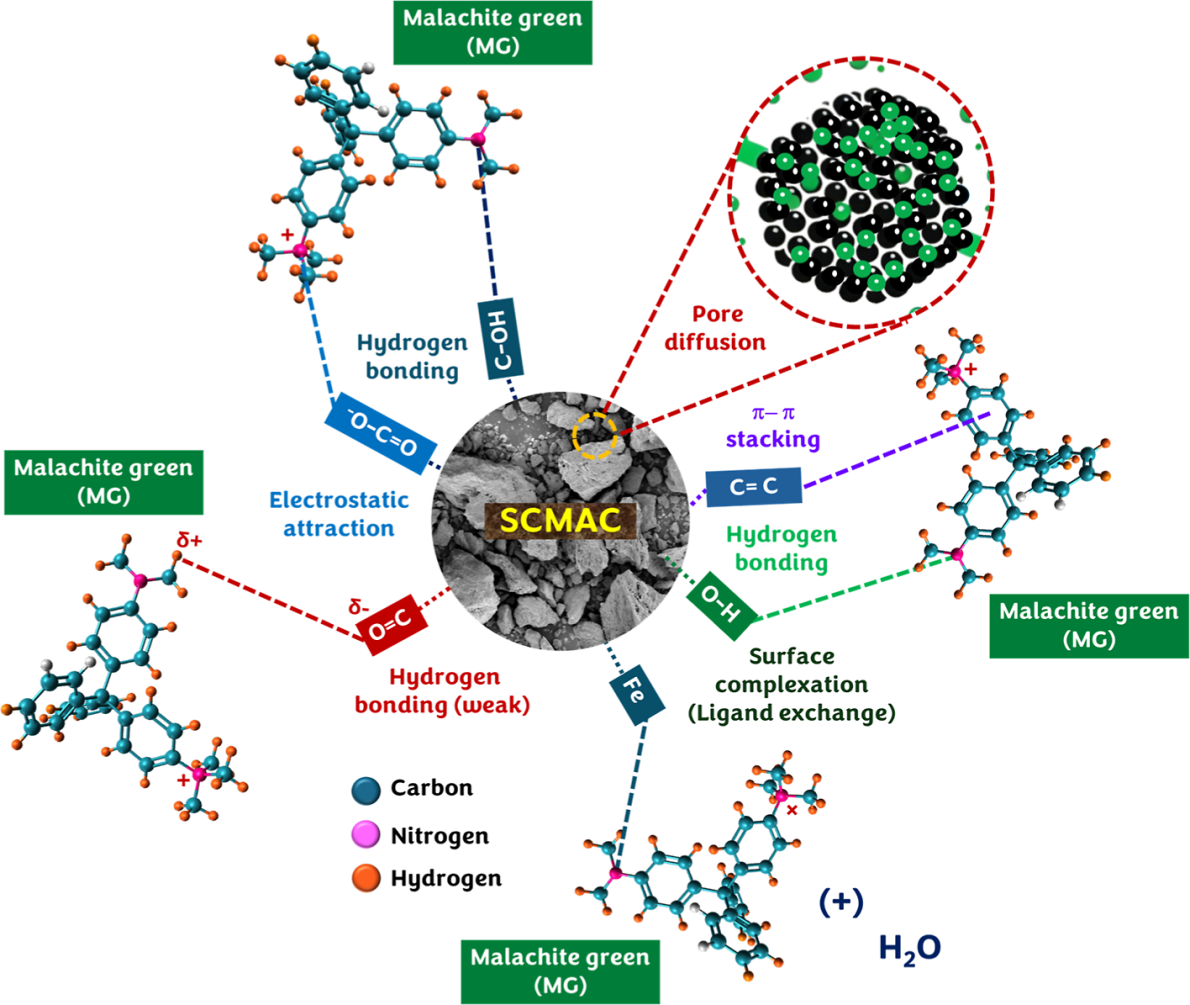
Schematic representation showing the key interactions between SCMAC and MG during adsorption (ball-and-stick 3D molecular structure of MG molecules was generated using Avogadro v1.2.0, a free and open-source molecular editor and visualization platform).

Further insights into surface interactions were provided by FTIR spectra, which showed distinct shifts in key vibrational bands upon MG adsorption. The O–H stretching vibration shifted slightly from 3896 to 3898 cm^–1^, and the C–O stretching band shifted from 939 to 945 cm^–1^, together indicating the involvement of surface hydroxyl (−OH) and phenolic (aromatic–OH) groups on SCMAC in hydrogen bonding interactions with the uncharged dimethylamino group (−N­(CH_3_)_2_) of the MG molecule. Supporting this interpretation, Lotha et al. reported a broad O–H stretching band at 3698 cm^–1^ during MG adsorption onto AC derived from pig hooves biomass (PHAC), attributing it to strong hydrogen bonding.^71^ Additionally, the C=C stretching band shifted from 1583 to 1587 cm^–1^, indicating the occurrence of π–π stacking interactions between MG’s aromatic rings and the graphitic domains of SCMAC. This finding aligns with the observations of Elwardany et al., who reported that π–π interactions between MG and the aromatic domains of guava seed-derived AC facilitated effective dye adsorption.^72^

Moreover, the Fe–O vibration band exhibited a shift from 505 to 509 cm^–1^, which can be attributed to weak coordination between MG molecules and Fe–O sites present in Fe_3_O_4_ embedded within SCMAC, suggesting the contribution of surface complexation (possibly via ligand exchange) to the overall adsorption mechanism. Affirming this mechanism, Altintig et al. investigated MG adsorption onto Fe_3_O_4_-modified AC derived from hazelnut shells and observed a characteristic Fe–O band within 500–750 cm^–1^.^29^ A slight shift in this band upon MG loading confirmed the involvement of Fe–O groups in surface complexation. These findings were further substantiated by XPS analysis, which revealed additional shifts: the C 1s (C–C/C=C) peak shifted from 284.44 to 284.64 eV, C–O from 286.11 to 286.23 eV, and O=C–O from 288.48 to 288.79 eV. Corresponding changes in the O 1s peaks were also observed, with the C=O and C–O components shifting from 531.76 to 532.04 eV and 529.94 to 530.34 eV, respectively. These spectroscopic shifts confirm the occurrence of π–π interactions as well as hydrogen bonding between SCMAC and the MG dye. In parallel, the Fe 2p 3/2 and Fe 2p 1/2 peaks moved from 710.78 to 711.58 eV and 724.70 to 725.06 eV, respectively, lending additional support to the proposed surface complexation or ligand exchange between MG molecules and Fe^2+^/Fe^3+^sites on Fe_3_O_4_.

Besides, the pH-dependent adsorption profile showed maximum MG removal at pH 6, where SCMAC, having a PZC of 3.5, is negatively charged, while MG (p*K*_a_ = 6.9) remains cationic, promoting electrostatic attraction. A similar observation by Ahmad et al. for AC derived from gasified *H. brasiliensis* roots confirmed enhanced MG adsorption at pH levels above the adsorbent’s PZC, highlighting the significance of electrostatic interactions.^49^ Kinetic modeling of the adsorption process followed the PSO model, indicating that chemisorption is the predominant rate-limiting step, although film diffusion and boundary layer effects may contribute during the early stages of adsorption. Equilibrium behavior was best captured by the Freundlich isotherm model indicating heterogeneous and multilayer adsorption possibilities. Thermodynamic parameters reinforced these findings: negative Gibbs free energy (Δ*G*°) values from 293–323 K confirmed spontaneity, the positive enthalpy change (Δ*H*° = 41.52 kJ/mol) indicated an endothermic process, and the substantial entropy change (Δ*S*° = 162.51 J/mol K) reflected increased randomness and enhanced affinity at the solid–liquid interface. Altogether, the adsorption of MG onto SCMAC proceeds through a synergistic mechanism involving electrostatic attraction, hydrogen bonding, π–π stacking and surface complexation within a highly porous, functionalized, and magnetically responsive framework.

## 3 Conclusion

This study demonstrated the successful synthesis and application of magnetic activated carbon derived from *S. campanulata* flowers for the effective removal of malachite green dye from aqueous solutions. The material, produced via phosphoric acid activation followed by Fe_3_O_4_ functionalization, exhibited a high surface area, mesoporous structure, and rich surface functionalities such as hydroxyl and carbonyl groups. These features contributed to a high adsorption capacity through multiple mechanisms, including electrostatic attraction, hydrogen bonding, π–π interactions, and surface complexation. Adsorption followed pseudo-second-order kinetics, indicating chemisorption as the dominant mechanism, while equilibrium data best fit the Freundlich isotherm model, reflecting multilayer adsorption on a heterogeneous surface. Thermodynamic analysis confirmed the spontaneous and endothermic nature of the process. The adsorbent retained significant efficiency over six reuse cycles and maintained consistent performance across various natural water samples. Furthermore, phytotoxicity studies confirmed that treated solutions were safe for seed germination and plant growth. These results establish *S. campanulata*-based magnetic carbon as a sustainable and environmentally friendly adsorbent for dye-contaminated wastewater. Future work should explore economic and life cycle assessments, continuous flow applications, performance evaluation in complex multicontaminant systems, and the inclusion of additional plant physiological and biochemical parameters such as biomass and chlorophyll content to support large-scale and sustainable deployment.

## 4 Materials and Methods

### 4.1 Materials

Malachite green (MG) dye (C_23_H_25_ClN_2_) was obtained from Himedia, India. Orthophosphoric acid (H_3_PO_4_, 85%) was procured from Sigma-Aldrich, USA, while sodium bicarbonate (NaHCO_3_) and ferrous sulfate heptahydrate (FeSO_4_·7H_2_O) were sourced from Merck, Germany. FeSO_4_·7H_2_O was chosen as a stable iron source due to its high solubility, affordability, and suitability for the formation of Fe_3_O_4_. Additionally, NaOH and HCl were purchased from Fisher Scientific, USA. Sodium hypochlorite (NaOCl) was acquired from Loba Chemie, India. Seeds of *S. lycopersicum* were purchased from Kurkal Agro Products, Udupi, India. Flowers of *S. campanulata* were gathered from the university campus in Manipal, India.

### 4.2 Methods

#### 4.2.1 Preparation of Adsorbent

AC was synthesized following a procedure, akin to what we described in our previous study.^7^ To begin, flowers from *S. campanulata* tree were cleaned, washed, and dried (oven, 80 °C, 12 h). Later, they were ground into a fine powder and combined with H_3_PO_4_ in a 1:1 (w/v) ratio for chemical activation and left idle for 6 h. After this, the blend was kept for aging (hot air oven, 80 °C, 12 h). The aged material was thereafter carbonized (muffle furnace, 400 °C, 2 h). Subsequently, the product was rinsed with NaHCO_3_ (1% w/v) solution until a neutral pH was attained. Lastly, the material was dried (oven, 100 °C, 12 h), resulting in the activated carbon, denoted as AC (Figure S3).

To synthesize magnetic activated carbon (MAC), 2.8 g of AC was combined with 1.4 g of FeSO_4_·7H_2_O (2:1 ratio) in 50 mL distilled water and stirred (150 rpm, 1 h, room temperature).^73^ Then, 10 mL of 2 M NaOH was introduced, and the solution was agitated for 15 min. Afterward, the mixture was heated (water bath, 80 °C, 1 h), after which the resulting magnetic material was extracted by a strong magnet. It was rinsed well with distilled water until neutral pH and dried (oven, 80 °C, 1 h). The final contents were designated as SCMAC and placed in an airtight container for future usage (Figure S3).

#### 4.2.2 Structural and Functional Analysis of *S. campanulata*

The porous nature of SCMAC was analyzed via nitrogen adsorption–desorption isotherms and Barrett–Joyner–Halenda (BJH) pore size distribution (Microtrac BELSORP-MAxX, Japan) methods. The morphological and elemental nature were assessed using field emission scanning electron microscopy (FESEM, Zeiss Sigma 300, Carl Zeiss, Germany) integrated with energy-dispersive X-ray spectroscopy (EDS, Oxford Instrument, UK). Fourier transform infrared spectroscopy (FTIR, Shimadzu 8400S, Japan) was utilized to examine the chemical moieties. The crystalline structure of SCMAC was analyzed by X-ray diffraction (XRD, D8 Advance, Bruker, Germany), while X-ray photoelectron spectroscopy (XPS, ThermoFisher, UK) was employed to assess the surface chemistry and bonding configurations. Furthermore, the magnetic behavior of SCMAC was quantified using a vibrating sample magnetometer (VSM, Lakeshore Cryotronics—VSM 8600 magnetometer). The point-of-zero charge (PZC) of SCMAC was determined using the pH drift method.^73^

#### 4.2.3 Adsorption Experiment Procedure

Adsorption tests were done to assess the efficiency of SCMAC in removing MG dye from aqueous solutions. The first step involved studying the influence of pH by adding 0.15 g/L of SCMAC into 100 mL MG dye (15 mg/L), with the pH adjusted between 3 and 8 (extreme pH conditions were not considered in this study, as MG undergoes structural color changes at pH 2 and significant degradation due to hydroxide ion reactions at pH 10–12). SCMAC dose effect was assessed by altering the dose between 0.05 and 0.25 g/L at optimal pH and 15 mg/L MG concentration. Consequently, experiments were performed to analyze the impact of the initial MG titer, spanning between 5 and 25 mg/L, and the contact time, using the optimized pH and SCMAC dosage. The impact of temperature was also examined by changing it between 293 and 323 K while keeping the ideal pH at 4.0, the SCMAC dose at 0.15 g/L, and the MG concentration at 15 mg/L. Throughout all experiments, the solutions were agitated at 150 rpm and 30 °C utilizing a thermostat-shaker (Remi CIS-24 Plus). Samples drawn at regular duration were monitored for residual MG concentrations with a UV–visible spectrophotometer (Shimadzu UV-1900i) at 617 nm. Samples with absorbance >1.0 were diluted with distilled water to ensure they fell within the linear calibration range (absorbance <1). Triplicate trials were performed to ensure reliability, and the mean values were presented. The removal efficiency (*R*, %) and adsorption capacity (*q*_e_, mg/g) were computed using the respective eqs 1 and 2.

1 $$R=\frac{C_{i}-C_{t}}{C_{i}}\times 100$$

2 $$q_{e}=\frac{\left(C_{i}-C_{e}\right)V}{M}$$

Here *C*_i_, *C*_t_, and *C*_e_ point to MG concentrations at the beginning, time ‘*t*’, and equilibrium (mg/L). *V* denotes volume (L), and ‘*M*’ represents the weight of SCMAC (g).

### 4.3 Adsorption Modeling Studies

Kinetic studies were conducted to elucidate the rate-controlling mechanism for MG adsorption onto SCMAC. The kinetic data were investigated using widely accepted model equations: pseudo-first-order (PFO), pseudo-second-order (PSO), and intraparticle diffusion (IPD). To assess the adsorption mechanism and performance of SCMAC for MG removal, the experimental data was modeled to adsorption isotherms, including Langmuir, Freundlich, and Temkin. The thermodynamic variables were calculated to evaluate the spontaneity, feasibility, and nature of the MG adsorption onto SCMAC.

### 4.4 Assessing the Effectiveness of *S. campanulata* to Remove Malachite Green Dye from Real Water Samples

Water samples from seven different sources, including Sai River (SI), Industrial Ground Water (IGW), Suvarna River (SR), Sarjoo River (SJ), Manipal Lake (ML), Ganga (GA), and Yamuna (YA) were collected through grab sampling. Distilled water (DW) was included as a control. IGW from a well at a textile site was chosen for its expected range of dissolved ions and contaminants typical of industrial wastewater and significantly different from natural water resources such as rivers and lakes. These samples were spiked with MG dye under optimum conditions to evaluate the adsorption performance of SCMAC in various real-world conditions.

### 4.5 Regeneration Ability of *S. campanulata*

Regeneration studies were executed to assess the reusability of spent SCMAC and enhance its economic feasibility. Absolute methanol was tested for its efficiency in removing MG from spent SCMAC. After adsorption experiments using a 15 mg/L MG solution and 0.15 g/L SCMAC, the spent adsorbent was impregnated with 50 mL of methanol and agitated for 3 h. Following the desorption process, the SCMAC was collected and dried (hot air oven, 80 °C, 2 h). To examine the reusability of SCMAC, six successive adsorption–desorption cycles were conducted. After every individual cycle, %*R* and *q*_e_ (mg/g) of the regenerated SCMAC were measured to determine its performance over repeated use.

### 4.6 Phytotoxicity Studies and Statistical Analysis

A pot-based phytotoxicity analysis was conducted using *S. lycopersicum* seeds following the modified protocol outlined by Das et al.^68^ Before their usage in the phytotoxic studies, the seeds were surface sterilized to eliminate potential microbial contamination. This was achieved by soaking the seeds in a 1% NaOCl solution for 30 min, preceded by rinsing three times with distilled water to eliminate any residual sterilizing agent. The experiment was divided into two parts: one involving MG solutions before adsorption (untreated) and the other using MG solutions after adsorption (treated). To assess the phytotoxic effects comprehensively, MG concentrations of 5, 15, and 25 mg/L were tested, with distilled water serving as the control to establish baseline growth conditions.

Pots were prepared using a homogenized mixture of soil and cocopeat in a 2:1 weight ratio to ensure optimal aeration and moisture retention. This substrate blend was uniformly mixed prior to use to maintain consistent nutrient content and texture across all replicates. Seven seeds of *S. lycopersicum* were sown uniformly in each pot at a consistent depth and spacing. In the “before adsorption” experiment, 5 mL of MG solutions at the designated concentrations were prepared and applied to the pots, while control pots received an equivalent volume of distilled water. Separately, MG solutions were treated with SCMAC under optimized adsorption conditions, and the treated solutions were collected and applied to the pots in the “after adsorption” experiment. All the experiments were performed in triplicates. To maintain consistent moisture levels, respective solutions were added to each pot twice daily at 12 h intervals over 7 days. All pots were maintained under controlled environmental conditions, with a temperature of 25 ± 2 °C, and simulated circadian rhythm through alternating light and dark cycles.^74^

On the seventh day, the germination index (GI, %), mean shoot length (SL, cm), as well as mean root length (RL, cm) were measured. The GI was calculated using the eq 3.

3 $$GI\;\left(\%\right)=\frac{number\;of\;seeds\;germinated}{total\;number\;of\;seeds\;sown}\times 100$$

The measured parameters were compared across the tested MG concentrations and the control to determine the phytotoxic effects of untreated and SCMAC-treated MG solutions. Statistical analyses were performed using Tukey’s test at a 5% significance level in OriginPro 2025.

## Supplementary Material



## Notes

Figure S1a,b shows the XRD patterns and FTIR spectra of SCMAC before and after malachite green (MG) adsorption, while Figure S2 depicts the point-of-zero charge (PZC) of the adsorbent. Figure S3 presents a schematic illustration of the synthesis process for the superparamagnetic activated carbon (SCMAC) derived from *S. campanulata* flowers. Table S1 provides a comparative overview of various activated carbons (AC) reported for MG dye removal, highlighting preparation conditions, BET surface areas, and maximum adsorption capacities (*q*_m_, mg/g) (PDF)
