# Supplementary material for: Phytotoxicity Mitigation and Malachite Green Removal from Wastewater Using Superparamagnetic Activated Carbon
Source: ACS Omega. 2025 Jul 21;10(30):33788–805. doi: 10.1021/acsomega.5c04838 (PMC12332674; doi:10.1021/acsomega.5c04838)
Supplement: Supplementary file 1 [file ao5c04838_si_001.pdf]

Phytotoxicity Mitigation and Malachite Green Removal from Wastewater Using Superparamagnetic Activated Carbon

Sujesh Sudarsan [a], Gokulakrishnan Murugesan [b],
Thivaharan Varadavenkatesan [c], Ramesh Vinayagam [a*], Raja Selvaraj [a*]

[a] Department of Chemical Engineering, Manipal Institute of Technology, Manipal Academy of Higher Education, Manipal, 576104, Karnataka, India

[b] Department of Biotechnology, M.S. Ramaiah Institute of Technology, Bengaluru, 560054, Karnataka, India

[c] Department of Biotechnology, Manipal Institute of Technology, Manipal Academy of Higher Education, Manipal, 576104, Karnataka, India

* Email: ramesh.v@manipal.edu; Phone: +91- 9742506638

* Email: raja.s@manipal.edu; Phone: +91- 9964582441

Supporting Information

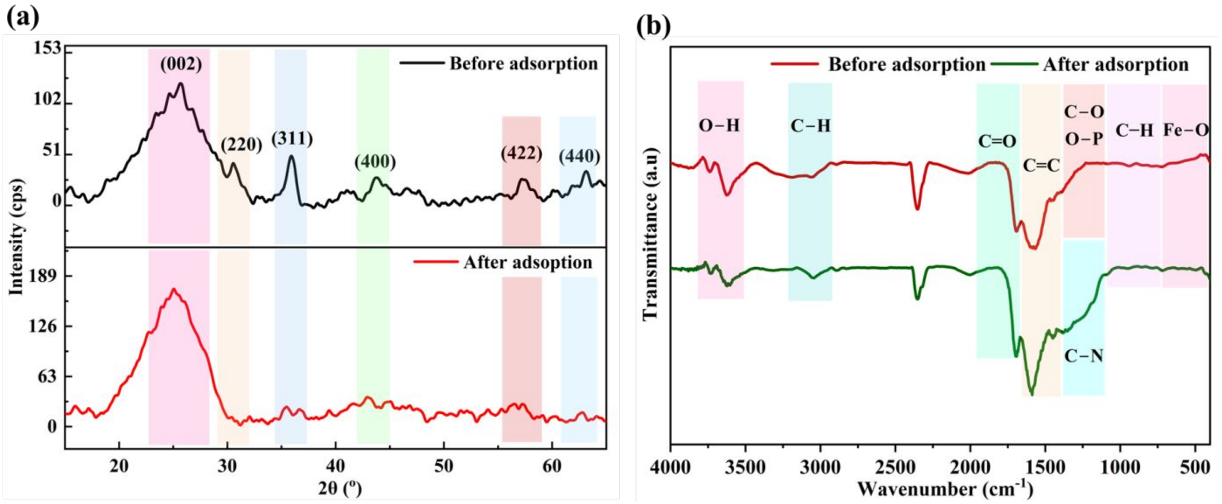

Figure S1 (a) XRD patterns of SCMAC before and after adsorption; **(b)** FTIR spectra of SCMAC before and after adsorption

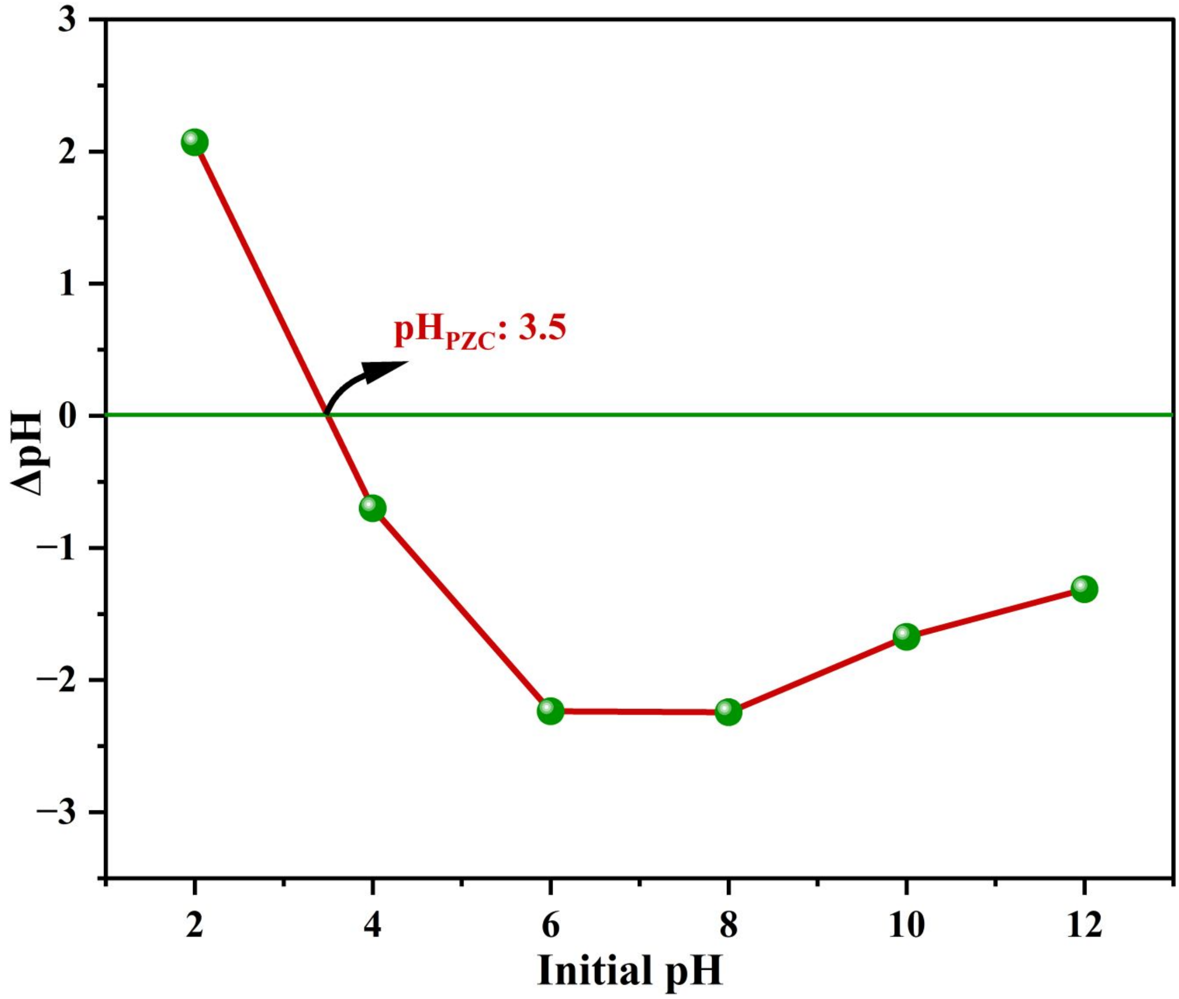

Figure S2. Point-of-zero charge (PZC) of SCMAC

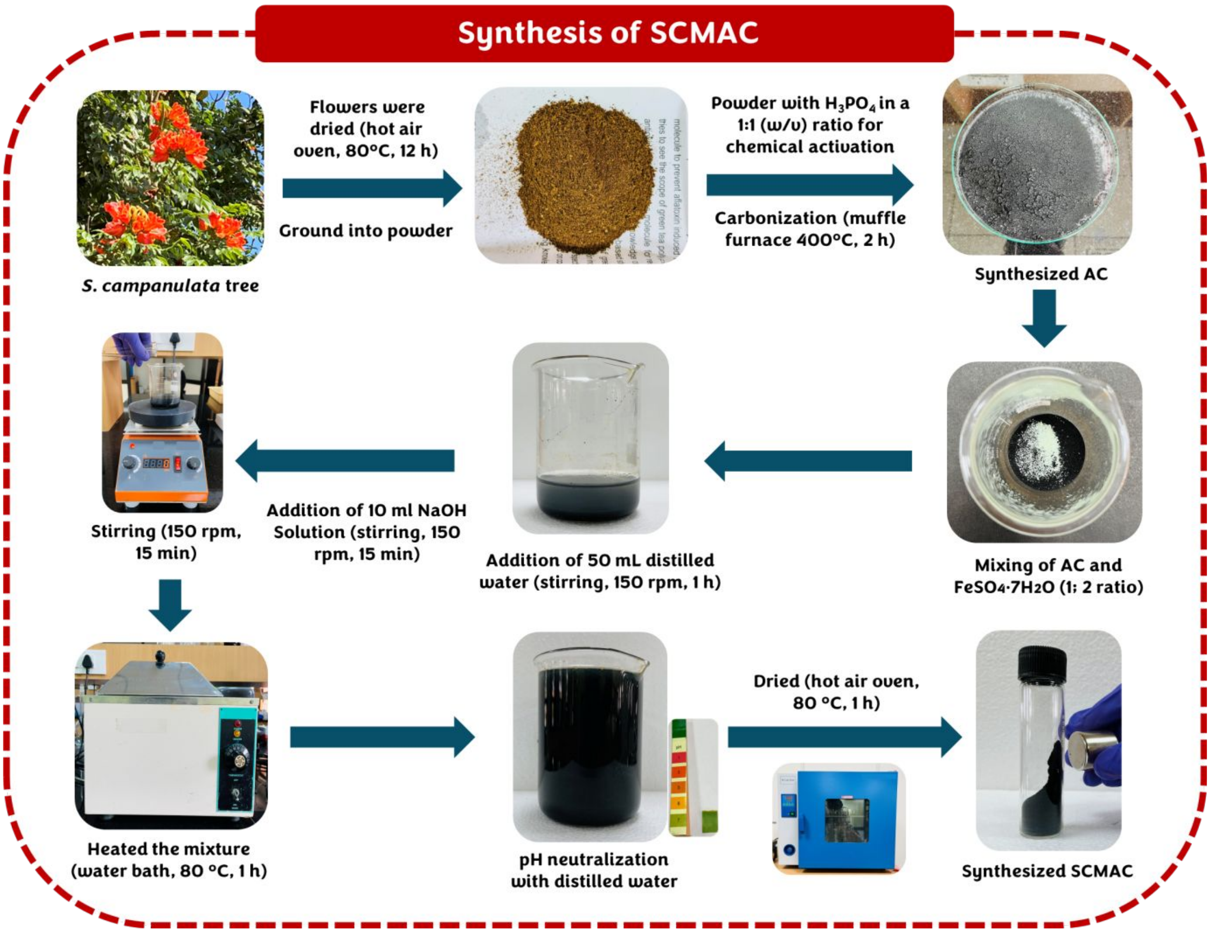

Figure S3. Schematic representation of the synthesis process for SCMAC derived from *S. campanulata* flowers

Table S1. Comparison of various activated carbon for MG dye removal

Sl. No	Adsorbent	AC preparation conditions	BET surface Area (m^2/g)	q_m (mg/g)	Ref.
1	*Catha edulis* stem AC	NaOH, 400 ^oC, 2h	1735	5.620	1
2	Pig Hooves AC	KOH, 700 °C, 3 hr	334.031	10.061	2
3	Granular AC	-	589.87	10.3413	3
4	Coconut shell AC	$ZnCl_2$, 550 °C, 1 h	223.77	17.90	4
5	Coconut shell carbon	100 °C for 2 h	75.2	18.87	5
6	Mangosteen peel AC	H_2SO_4, 150 °C, 24 h	-	19.345	6
7	*Psidium guajava AC*	KOH ,600 °C, 3 h	905.27	24.55	7
8	Coconut shell AC	H_3PO_4, 110 ℃, 12 h	419.70	32.787	8
9	*Ziziphus spina*-christi AC	H_2SO_4, 200 ^oC, 4 h	21.49	48.78	9
10	*Hevea brasiliensis* seed Shells AC	H_3PO_4, 300 °C, 3 h	999	58.48	10
11	*Spathodea campanulata* derived MAC (SCMAC)	H_3PO_4, 400 ^oC, 2 h	1012.9	72.73	This study

References

(1) Abate, G. Y.; Alene, A. N.; Habte, A. T.; Getahun, D. M. Adsorptive Removal of Malachite Green Dye from Aqueous Solution onto Activated Carbon of Catha Edulis Stem as a Low Cost Bio-Adsorbent. *Environmental Systems Research* **2020**, *9* (1), 29. https://doi.org/10.1186/s40068-020-00191-4.

(2) Lotha, T. N.; Rituparna, K.; Vevosa, N.; Lemzila, R.; Francis A. S., C.; and Jamir, L. Valorizing Pig Hooves for Activated Carbon Production with Efficient Methyl Green Adsorption, DFT Insights and Bacterial Removal via Bio-Adsorbent Column. *Green*

Chemistry Letters and Reviews **2024**, *17* (1), 2431261. https://doi.org/10.1080/17518253.2024.2431261.

(3) Mohammad, M.; Maitra, S.; Dutta, B. K. Comparison of Activated Carbon and Physic Seed Hull for the Removal of Malachite Green Dye from Aqueous Solution. *Water, Air, & Soil Pollution* **2018**, *229* (2), 45. https://doi.org/10.1007/s11270-018-3686-4.

(4) Sangeetha Piriya, R.; Jayabalakrishnan, R. M.; Maheswari, M.; Boomiraj, K.; Oumabady, S. Coconut Shell Derived ZnCl2 Activated Carbon for Malachite Green Dye Removal. *Water Science and Technology* **2021**, *83* (5), 1167–1182.

(5) Mishra, S. P.; Patra, A. R.; Das, S. Methylene Blue and Malachite Green Removal from Aqueous Solution Using Waste Activated Carbon. *Biointerface Research in Applied Chemistry* **2021**, *11* (1), 7410–7421.

(6) Yuningsih, N. E.; Ariani, L.; Suprapto; Ulfin, I.; Harmami; Juwono, H.; Ni'mah, Y. L. Adsorption of Malachite Green Using Activated Carbon from Mangosteen Peel: Optimization Using Box-Behnken Design. *Journal of Renewable Materials* . 2024. https://doi.org/10.32604/jrm.2024.049109.

(7) Elwardany, R. E.; Shokry, H.; Mustafa, A. A.; Ali, A. E. Influence of the Prepared Activated Carbon on Cellulose Acetate for Malachite Green Dye Removal from Aqueous Solution. *Macromolecular Research* **2023**, *31* (11), 1043–1060. https://doi.org/10.1007/s13233-023-00187-w.

(8) Piriya, R. S.; Jayabalakrishnan, R. M.; Maheswari, M.; Boomiraj, K.; Oumabady, S. Comparative Adsorption Study of Malachite Green Dye on Acid-Activated Carbon. *International Journal of Environmental Analytical Chemistry* **2023**, *103* (1), 16–30.

(9) Saleh Bashanaini, M. Removal of Malachite Green Dye from Aqueous Solution by

Adsorption Using Modified and Unmodified Local Agriculture Waste. *Science Journal of Analytical Chemistry* **2019**, *7* (2), 42. https://doi.org/10.11648/j.sjac.20190702.12.

(10) Igwegbe, C. A.; Ighalo, J. O.; Onyechi, K. K.; Onukwuli, O. D. Adsorption of Congo Red and Malachite Green Using H3PO4 and NaCl-Modified Activated Carbon from Rubber (Hevea Brasiliensis) Seed Shells. *Sustainable Water Resources Management* **2021**, *7* (4), 63. https://doi.org/10.1007/s40899-021-00544-6.
